# Metalloproteinase inhibitors regulate biliary progenitor cells through sDLK1 in organoid models of liver injury

**DOI:** 10.1172/JCI164997

**Published:** 2024-12-19

**Authors:** Virginie Defamie, Kazeera Aliar, Soumili Sarkar, Foram Vyas, Ronak Shetty, Swami Reddy Narala, Hui Fang, Sanjay Saw, Pirashaanthy Tharmapalan, Otto Sanchez, Jennifer J. Knox, Paul D. Waterhouse, Rama Khokha

**Affiliations:** 1 Princess Margaret Cancer Centre, University Health Network, Toronto, Ontario, Canada.; 2 Department of Medical Biophysics, University of Toronto, Toronto, Ontario, Canada.; 3 Ontario Tech University, Oshawa, Ontario, Canada.; 4 Department of Medical Oncology and Hematology, Princess Margaret Cancer Centre, University of Toronto, Toronto, Ontario, Canada.

**Keywords:** Hepatology, Stem cells, Adult stem cells, Molecular biology, Proteases

## Abstract

Understanding cell fate regulation in the liver is necessary to advance cell therapies for hepatic disease. Liver progenitor cells (LPCs) contribute to tissue regeneration after severe hepatic injury, yet signals instructing progenitor cell dynamics and fate are largely unknown. Tissue inhibitor of metalloproteinases 1 (TIMP1) and TIMP3 control the sheddases ADAM10 and ADAM17, key for NOTCH activation. Here we uncover the role of the TIMP/ADAM/NOTCH/DLK1 axis in LPC maintenance and cholangiocyte specification. Combined TIMP1/TIMP3 loss in vivo caused abnormal portal triad stoichiometry accompanied by collagen deposits, dysregulated Notch signaling, and increased soluble DLK1. The MIC1-1C3^+^CD133^+^CD26^–^ biliary progenitor population was reduced following acute CCl_4_ or chronic DDC liver injury and in aged TIMP-deficient livers. Single-cell RNA sequencing data interrogation and RNAscope identified portal mesenchymal cells coexpressing ADAM17/DLK1 as enzymatically equipped to process DLK1 and direct LPC differentiation. Specifically, TIMP-deficient biliary fragment–derived organoids displayed increased propensity for cholangiocyte differentiation. ADAM17 inhibition reduced *Sox9*-mediated cholangiocyte differentiation, prolonging organoid growth and survival, whereas WT organoids treated with soluble DLK1 triggered *Sox9* expression and cholangiocyte specification in mouse and patient-derived liver organoids. Thus, metalloproteinase inhibitors regulate instructive signals for biliary cell differentiation and LPC preservation within the portal niche, providing a new basis for cell therapy strategies.

## Abstract 2

Natural protease inhibitors oversee local sDLK1 bioavailability in the liver progenitor niche whereby mesenchymal cells trigger cell differentiation through increased Notch and sDLK1 signaling effectors.

## Graphical abstract

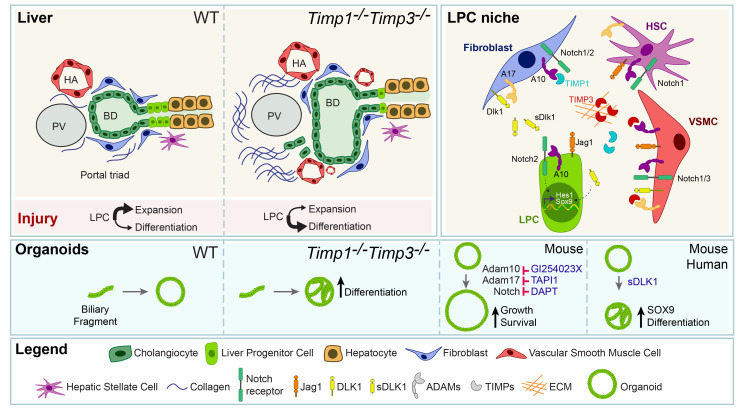

## Introduction

Orthotopic liver transplantation is the only curative approach for patients suffering from acute liver failure or chronic liver disease. Progress in stem cell biology is opening new possibilities for alternative therapies. Although hepatocytes have extensive replicative capacity, evidence indicates that the biliary compartment plays a crucial role in regeneration following extensive hepatocyte loss (1, 2). Biliary cell subpopulations comprise bipotent progenitor cells capable of generating both hepatocytes and cholangiocyte lineages (2, 3). Numerous studies have aimed to define liver progenitor cells (LPCs) and their contribution to hepatic homeostasis and repair, yet factors that influence LPC dynamics remain largely obscure.

The Notch pathway is a master regulator of cell fate, and its functions are well established in liver development. Notch is critical for the specification and differentiation of hepatoblasts into cholangiocytes (4, 5) and for morphogenesis of the intrahepatic biliary system (6, 7). The Notch ligand JAG1 along with NOTCH2 is instrumental for bile duct formation (8), and mutation of *JAG1* leads to bile duct paucity in Alagille syndrome in humans (9). Beyond its developmental role, Notch signaling participates in liver regeneration, fibrosis, metabolism, and cancer (10). Following ligand binding (Delta-like 1, 3, 4, Jagged 1, 2), Notch receptor activation (NOTCH1–4) requires metalloproteinase (ADAM)-mediated S2 cleavage of the receptor at the cell surface with subsequent NICD release via γ-secretase (S3 cleavage) (11). ADAM10 is firmly established as the central metalloproteinase responsible for Notch activation, and ADAM17 has also been proposed to perform this function (12, 13). Among Notch ligands, isoforms of Delta-like 1 homolog (DLK1; also called Pref1/FA1) are either membrane-tethered or processed by ADAM17, which releases its soluble form (sDLK1) (14, 15). Wide embryonic DLK1 expression becomes restricted to a few tissues or stem/progenitor cells postnatally (16). In the human and mouse embryonic liver, DLK1 serves as a marker for bipotent hepatoblasts (17, 18) and is expressed by rat oval cells in severely injured tissue (19). Among the 4 natural tissue inhibitors of metalloproteinases, TIMP1 controls ADAM10 while TIMP3 inhibits both ADAM10 and ADAM17 activities (20, 21). Compound *Timp1*/*Timp3*-deficient mice exhibit striking mammary stem cell expansion, which we have shown prevents age-associated tissue regression in part via altered Notch activity (22). The TIMP-metalloproteinase axis is thus a fundamental regulator of Notch signaling (23, 24).

Here we uncover that TIMP1 and TIMP3 together control the mouse liver progenitor reservoir and highlight portal mesenchymal cells as regulators of LPC fate. Using biliary organoid models, we demonstrate the increased propensity of LPCs toward cholangiocyte differentiation in a microenvironment lacking these TIMPs. Mechanistically, in addition to Notch activation we identify sDLK1 as an important inducer of *Sox9* and a trigger for cholangiocyte specification, revealing its critical function in the LPC niche. Exploiting small-molecule inhibitors, we show that control of ADAM activity is key to preserving LPC expansion and restricting cholangiocyte differentiation.

## Results

### Abnormal portal triad stoichiometry and biliary differentiation in compound TIMP1/TIMP3-null mice.

**Figure 1:**
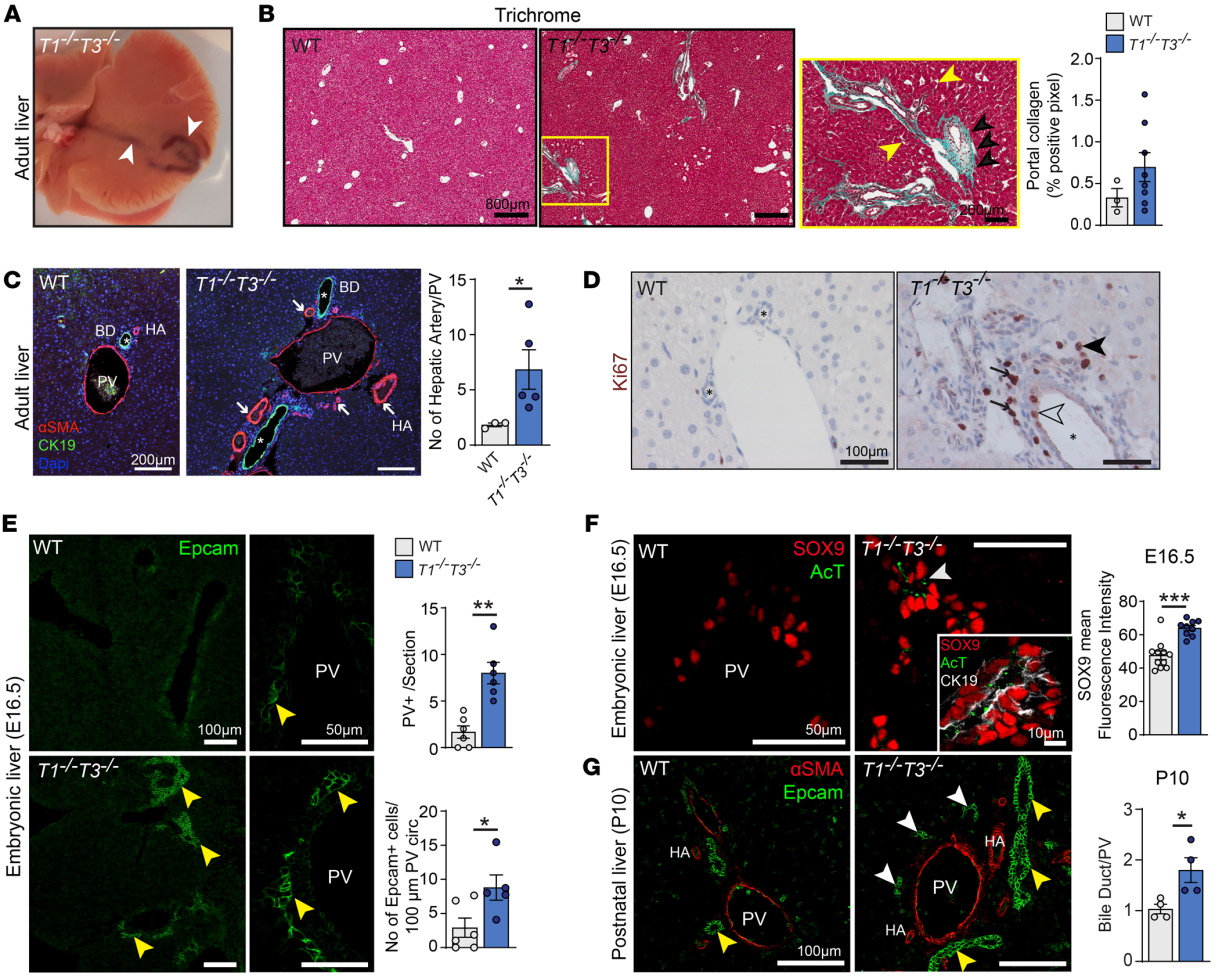
Distorted portal triad and excessive biliary differentiation in *Timp1^−⁄−^Timp3^−⁄−^* liver. (**A**) *Timp1^−⁄−^Timp3^−⁄−^* (*T1^−⁄−^T3^−⁄−^*) liver exhibiting abnormal superficial blood vessels (arrowheads). (**B**) Masson’s trichrome staining of liver sections; magnified area shows periportal collagen deposition in blue (black arrowheads) and sinusoid dilatation (yellow arrowheads). Portal collagen quantification; percentage of blue area on total tissue area. WT *n* = 3, *T1^−⁄−^T3^−⁄−^* *n* = 8 livers. (**C**) CK19^+^ immunofluorescence shows cholangiocytes forming bile ducts (BD; asterisks); αSMA^+^ smooth muscle cells form hepatic arteries (HA) and portal veins (PV). Arrows show abnormal periportal HA formation. Quantification of hepatic arteries per portal vein. WT *n* = 3, *T1^−⁄−^T3^−⁄−^* *n* = 5 livers. Mann-Whitney test, *P < 0.05. (**D**) Ki67 immunostaining highlights proliferating cholangiocytes (open arrowhead) in bile ducts (asterisks), interstitial cells (black arrows), and hepatocytes (black arrowhead) in *T1^−⁄−^T3^−⁄−^* portal area. (**E**) Immunofluorescence showing biliary duct formation. At E16.5, portal veins are surrounded by a single layer of Epcam^+^ cholangiocytes (yellow arrowheads) in WT, and a double layer of Epcam^+^ cells forming primitive ductal structures in *T1^−⁄−^T3^−⁄−^* embryos. Quantification of number of portal veins surrounded by Epcam^+^ cells in longitudinal sections of E16.5 livers (*n* = 6 livers per group) and number of Epcam^+^ cells per portal vein circumference. WT *n* = 6, *T1^−⁄−^T3^−⁄−^* *n* = 5 portal veins per genotype. Mann-Whitney test, *P < 0.05, **P < 0.01. (**F**) Immunofluorescence for SOX9, acetylated tubulin (AcT), and CK19 highlights mature duct cells. Data represent the average of the mean of mean fluorescence intensity of periportal SOX9^+^ nuclei. WT 11, *T1^−⁄−^T3^−⁄−^* 9 portal veins per genotype. Mann-Whitney test, ***P < 0.001. (**G**) Ten days after birth, Epcam^+^ cholangiocytes belong to bile ducts (yellow arrowheads) or are found in clusters scattered in the periportal area (white arrowheads). αSMA^+^ VSMCs highlight portal vein (PV) and arteries (HA). Bile duct/PV ratio at 10 days after birth (P10); *n* = 4 livers per genotype. Mann-Whitney test, *P < 0.05.

Mouse livers from individual or combined whole-body deletions of *Timp1 and Timp3* were analyzed (Supplemental Figure 1A; supplemental material available online with this article; https://doi.org/10.1172/JCI164997DS1). At the gross anatomical level, *Timp1^−⁄−^Timp3^−⁄−^* (designated hereafter as *T1^−⁄−^T3^−⁄−^*) livers were enlarged, of harder consistency and abnormal shape with visible large intrahepatic and portal blood vessels (Figure 1A), whereas single-knockout livers were similar to wild type (WT). Features of portal hypertension including hepatomegaly, splenomegaly, and numerous enlarged α-smooth muscle actin–positive (αSMA^+^) mesenteric blood vessels were noted in compound-knockout mice, but not individual *T1^−⁄−^* or *T3^−⁄−^* groups (Supplemental Figure 1, B–D). Structurally, *T1^−⁄−^T3^−⁄−^* liver also had evident lobular periportal collagen deposition (Figure 1B and Supplemental Figure 2A), and second-harmonic generation revealed discontinuous periportal collagen signals (Supplemental Figure 1E). Furthermore, the cholangiocyte marker cytokeratin 19 (CK19) and αSMA for vascular smooth muscle cells (VSMCs) confirmed altered portal triad stoichiometry, with elongated bile ducts surrounding the portal veins accompanied by a higher number of hepatic arteries (Figure 1C). At the cellular level, increased proliferation was evident in ductular and interstitial portal cells (Figure 1D), by Ki67 as well as proliferating cell nuclear antigen (PCNA) positivity (Supplemental Figure 1F). Overall, the severity of abnormalities increased with age, in spite of mouse-to-mouse variability, with highly proliferative bile ducts, extensive portal and sinusoid dilatation, and localized periportal fibrosis in 8-month-old *T1^−⁄−^T3^−⁄−^* mice (Supplemental Figure 3, A–E).

Developmentally, hepatic arteries form in response to signals from the bile ducts (25), and developmental anomalies in human polycystic liver disease have correlated increased hepatic artery branching with the presence of biliary cysts (26). Interrogation of published fetal liver single-cell RNA sequencing (scRNA-Seq) data (27) in human and mouse (human weeks 5–19; mouse E11.0–E17.5) revealed 8 major cell populations: hepatoblast/hepatocyte, cholangiocyte, erythroid lineage, hematopoietic lineage, Kupffer cells, mesenchymal cells (septum transversum stellate cells and mesothelial cells), and endothelial cells in both species. *Timp1* was expressed by mesenchymal and endothelial cells and *Timp3* by endothelial cells in embryonic livers (Supplemental Figure 4). At embryonic day 14.5 (E14.5), livers of WT and *T1^−⁄−^T3^−⁄−^* genotypes were comparable. (Sex-determining region Y)-box 9 (SOX9), an early marker of biliary cell differentiation downstream of Notch signaling, was present in periportal hepatoblasts, and the biliary cell marker Epcam was scarcely observed (Supplemental Figure 1G). However, *T1^−⁄−^T3^−⁄−^* liver at E16.5 had many portal veins surrounded by a greater number of Epcam+ cells (Figure 1E). Specifically, ductal structures were already formed in *T1^−⁄−^T3^−⁄−^* livers, while WT livers showed single-cell layers without ducts. Nascent tubules in *T1^−⁄−^T3^−⁄−^* hepatic tissue expressed markers of mature (CK19) and functional cholangiocytes (acetylated tubulin [AcT]) along with a significantly higher SOX9 fluorescence intensity (Figure 1F). At postnatal day 10 (P10), Epcam^+^ cells belonging to elongated biliary ducts were twice as numerous as in WT, and multiple single layers of Epcam^+^ cells were additionally found scattered in the *T1^−⁄−^T3^−⁄−^* periportal area (Figure 1G). This increase in bile duct number was less pronounced in adult tissue (Supplemental Figure 1H), suggesting attrition or lack of maintenance of bile ducts with aging. Altogether, the combined loss of TIMP1 and TIMP3 resulted in potent cholangiocyte differentiation during embryogenesis with subsequent aberrations in the adult hepatic portal triad architecture, suggesting that TIMP1 and TIMP3 are regulatory effectors of biliary lineage commitment.

### Increased Notch activation in the absence of TIMP1 and TIMP3.

**Figure 2:**
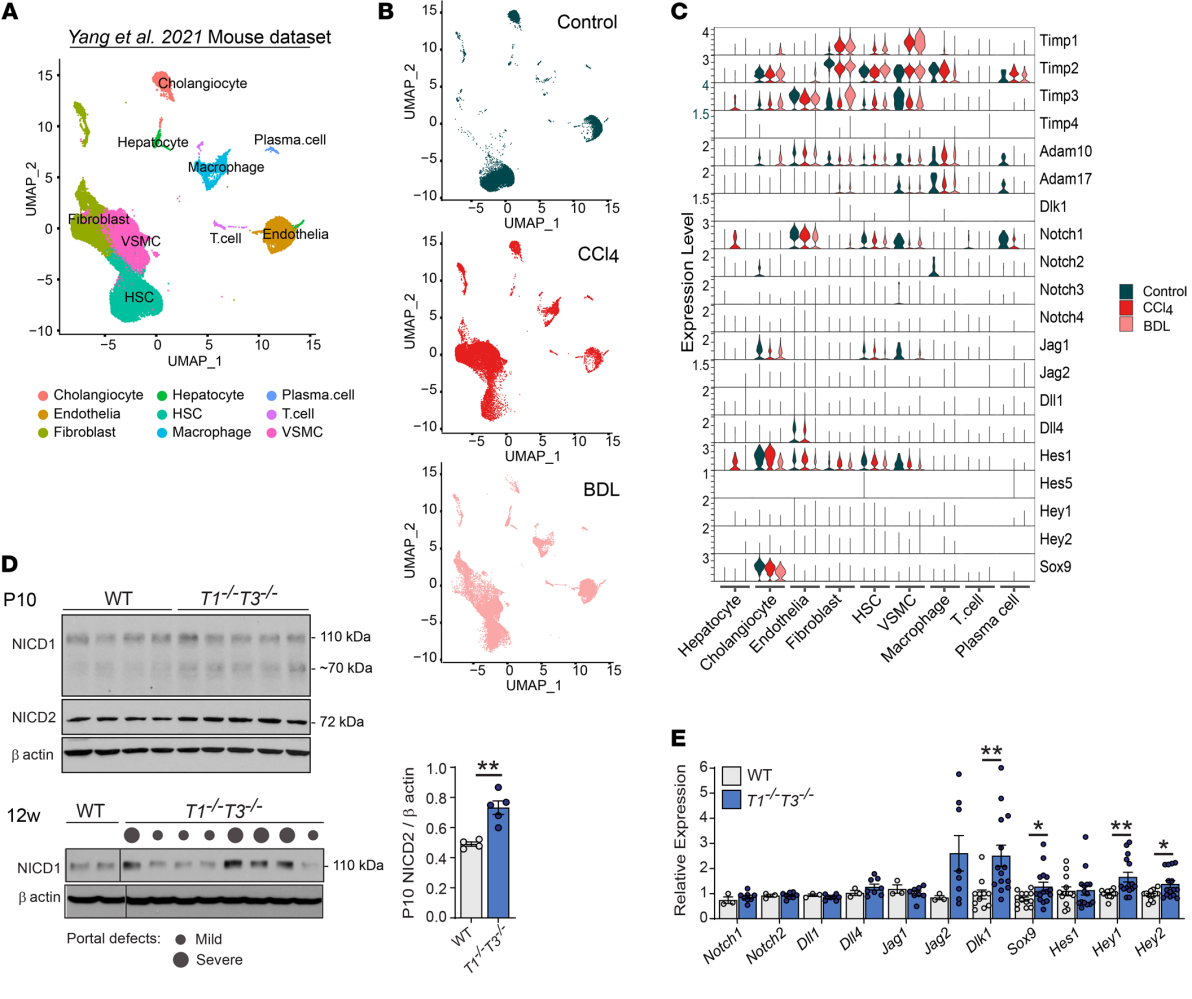
Increased Notch activation in the absence of TIMP1 and TIMP3. (**A**) UMAP visualization of isolated hepatic cells, based on 47,752 single-cell transcriptomes pooled from control (10,636), CCl_4_-treated (18,185), and bile duct–ligated mice (18,931) from the Yang et al. 2021 mouse dataset (28). (**B**) UMAP obtained for each condition. (**C**) Violin plots showing the expression of Timp, Adam, Notch ligands, receptors, and target genes in the hepatic cell populations defined in **A**, in control, CCl_4_-treated, and after bile duct ligation (BDL). (**D**) Western blot for cleaved Notch1 (NICD1) and Notch2 (NICD2; band density quantification, 2-tailed Student’s *t* test) in postnatal (P10) livers, and in 12-week-old livers. Notch1 cleavage correlates with severity of portal triad alterations (size of the dot) observed by Masson’s trichrome staining depicted in Figure 1B (based on collagen deposition and portal triad distortion). Black lines delineate lanes that were run on the same gel but were non-contiguous. (**E**) Expression of Notch target genes (*n* ≥ 11 livers) and Notch receptors and ligands (*n* ≥ 3 livers) in 12-week-old mice. *P < 0.05, **P < 0.01, 2-tailed Student’s *t* test. For Western blots, each lane represents 1 animal.

We next interrogated the published scRNA-Seq (Figure 2, A–C) of murine hepatic models of chronic injury after CCl_4_ exposure or bile duct ligation, both of which instigate LPC activation (28). TIMPs were broadly detectable in hepatic cells, except *Timp4* (Figure 2C). Cholangiocytes expressed *Timp2* and *Timp3*, while mesenchymal cells (hepatic stellate cells, fibroblasts, and VSMCs) expressed *Timp1*, *Timp2*, and *Timp3*. TIMP1, a known marker of liver fibrosis (29), was highly induced in mesenchymal cells after hepatic injury. Among ADAMs targeted by these TIMPs, *Adam10* was ubiquitous, while *Adam17* was found in macrophages, VSMCs, and fibroblasts. Components of the Notch pathway, Notch receptors 1–4 and ligands *Jag1*, *Jag2*, *Dll1*, *Dll4*, and *Dlk1*, were broadly detectable and the canonical target gene *Hes1* was present in cholangiocytes and endothelial and mesenchymal cells. *Sox9* was restricted to biliary cells. Next, direct examination of Notch signaling in our mouse cohorts by Western blotting found it was consistently increased in *T1^−⁄−^T3^−⁄−^* hepatic tissue. The activated form of Notch2 (NICD2) was elevated at P10 (Figure 2D), and a higher NICD1 level was obvious in adult liver (Figure 2D, 12w). Interestingly, the level of cleaved Notch1 correlated with the severity of portal triad distortion and collagen deposition (Figure 1B). Reverse transcription PCR (RT-PCR) analysis confirmed the upregulation of several Notch target genes (*Sox9*, *Hey1*, and *Hey2*) in *T1^−⁄−^T3^−⁄−^* adult liver. Expression of Notch receptors (1 and 2) and most ligands (*Dll1*, *Dll4*, *Jag1*, *Jag2*) was comparable to WT, but *Dlk1* was significantly increased in mice lacking these TIMPs (Figure 2E).

We also examined several morphogens and signaling pathways known to contribute to liver pathophysiology, specifically YAP, TGF-β, TNF-α, HGF, PDGF-B, and Hedgehog. Phosphorylation of YAP and ERK is important for cholangiocyte proliferation (30). Although p-ERK was elevated in *T1^−⁄−^T3^−⁄−^* livers, neither YAP nor its targets *Ccn1* and *Ccn2* were significantly altered (Supplemental Figure 5, A and B). A concentration gradient of transforming growth factor-β (TGF-β) initiates hepatoblast commitment toward biliary lineage (31), and inactive TGF-β precursor sequestered by the extracellular matrix is proteolytically activated by metalloproteinases (32). TGF-β activity was comparable at P10 (Supplemental Figure 5C) and at 12 weeks but increased at 8 months (Supplemental Figure 5D), likely owing to the advanced tissue alterations present in aged *T1^−⁄−^T3^−⁄−^* mice and correlating with TGF-β’s profibrotic role. Two other key regulators of liver cell proliferation and inflammation, hepatocyte growth factor (HGF) and tumor necrosis factor-α (TNF-α), which can be affected by TIMP activity (33, 34), were also measured (Supplemental Figure 5, E and F). While HGF levels were similar, TNF-α was elevated in *T1^−⁄−^T3^−⁄−^* livers. Given that hepatic stellate cells (HSCs) play a critical role in liver fibrosis, we measured HSC inducers and activation state. PDGF-B (HSC mitogen), *Des* and *Gfap* (HSC quiescence markers), and *Gli1*, *Gli2*, *Patch*, *Ihh*, and *Smo* (Hedgehog pathway involved in HSC activation) (35) were all comparable in adult WT and *T1^−⁄−^T3^−⁄−^* livers (Supplemental Figure 5, G and H). Collectively, these data pointed to overactive Notch signaling as a main driver of hepatic abnormalities in *T1^−⁄−^T3^−⁄−^* mice while ruling out the participation of YAP, TGF-β, HGF, PDGF-B, and Hedgehog pathways in *T1^−⁄−^T3^−⁄−^* hepatic alterations.

### Depletion of LPC pool after liver injury in T1^−⁄−^T3^−⁄−^ mice.

**Figure 3:**
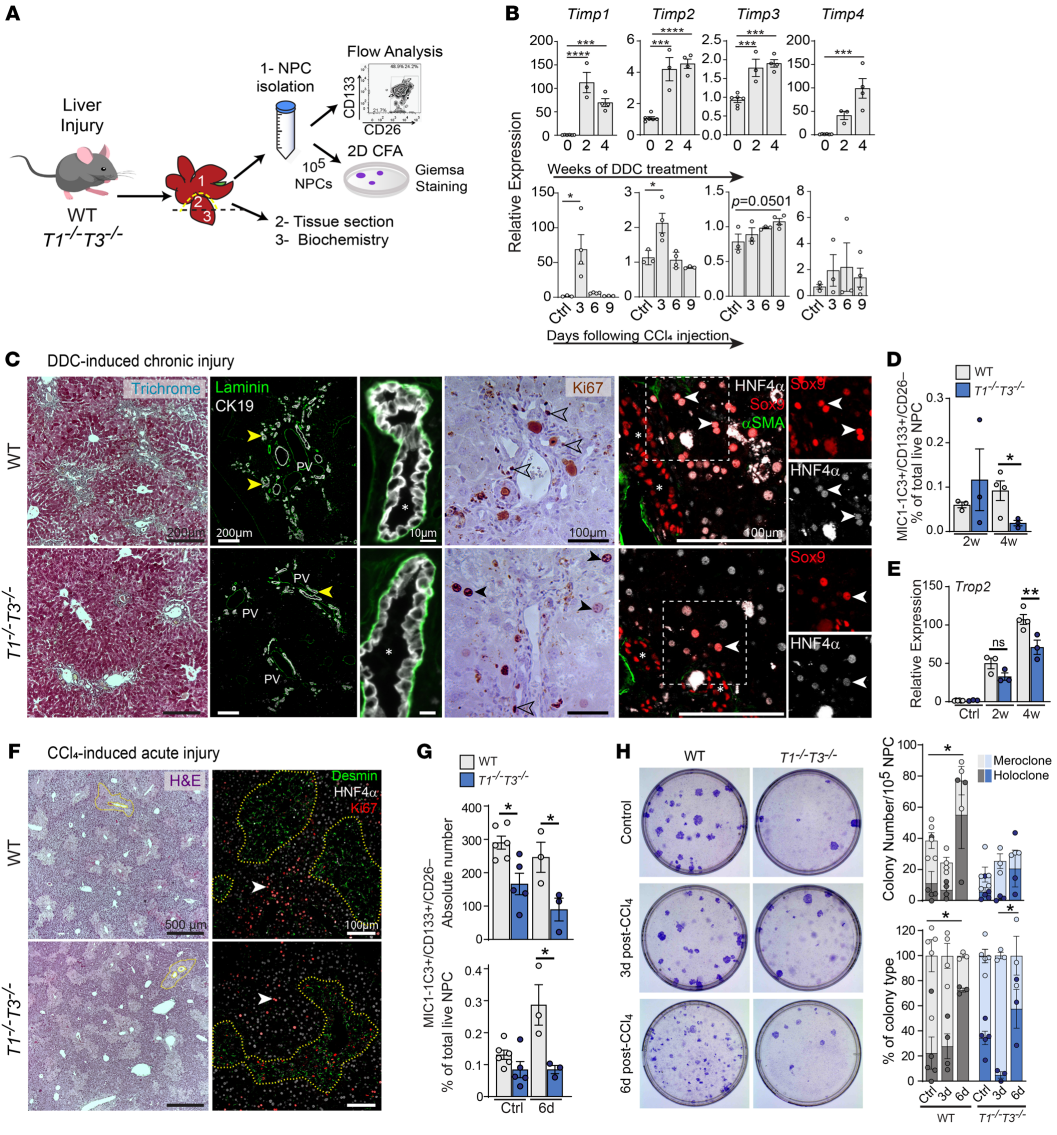
Impaired LPC expansion after liver injury. (**A**) Schematic of tissue analyses following 0.1% DDC– and CCl_4_-induced liver injury. Left hepatic lobe was harvested (yellow dashed line) for biochemistry and tissue staining, and the remaining organ was digested to obtain a single non-parenchymal cell (NPC) suspension for flow cytometry analysis. For CCl_4_-treated livers, NPCs were flow-profiled or cultured in CFC assay. (**B**) *Timp* expression in WT hepatic tissue following DDC or CCl_4_ administration relative to uninjured liver; *n* ≥ 3 livers per time point. One-way ANOVA with Šidák’s multiple-comparison test. (**C**) Liver tissue characterization after 4 weeks of DDC treatment. Fibrosis assessed with Masson’s trichrome staining; collagen deposition appears in blue. Ductular reaction (yellow arrowheads) seen by immunofluorescence for CK19 and laminin. Ki67 marks proliferative cholangiocytes (open arrowheads) and hepatocytes (black arrowheads). Immunofluorescence for specific markers (HNF4α, hepatocytes; SOX9, cholangiocytes; αSMA, VSMCs) within the ductular reaction. White arrowheads point to double-positive SOX9^+^HNF4α^+^ cells in the vicinity of bile ducts (asterisks). (**D** and **E**) Flow cytometry analysis of MIC1-1C3^+^CD133^+^CD26^–^ population (**D**) and whole-tissue expression of *Trop2* (**E**) after DDC treatment; *n* ≥ 3 livers. Two-way ANOVA with Tukey’s multiple-comparison test. (**F**) Characterization of WT and *T1^−⁄−^T3^−⁄−^* livers 3 days after CCl_4_ insult. H&E staining showing pericentral necrotic area (yellow dashed lines). Immunofluorescence for Ki67 shows proliferative hepatocytes in zone 1/2 (white arrowheads) and desmin-positive mesenchymal cell migration within the pericentral necrotic area. (**G**) Flow cytometry analysis of MIC1-1C3^+^CD133^+^CD26^–^ population at 6 days after CCl_4_ injury; *n* ≥ 3 livers per genotype per time point. Two-way ANOVA with Tukey’s multiple-comparison test. (**H**) Representative plates of colony-forming assay from untreated and post-CCl_4_ livers. Colony quantification graphed as absolute number of colonies and percentage of colony type (*n* ≥ 3). Two-way ANOVA with Tukey’s multiple-comparison test, *P < 0.05, **P < 0.01, ***P < 0.001, ****P < 0.0001.

LPCs are bipotent cells found at the hepatocytic-biliary interface in the canal of Hering (36), the region most affected by the combined loss of TIMP1 and TIMP3. Although hepatocytes are recognized as the major drivers of liver repair after damage (37, 38), LPC expansion has been reported in several common models of liver injury (3, 39). We therefore determined whether TIMP deletion affects biliary cell progenitor composition and fate by subjecting mice to (a) a chronic DDC (0.1% 3,5-diethoxycarbonyl-1,4-dihydrocollidine) regimen that elicits a ductular reaction (DR) associated with biliary progenitor activation, or (b) an acute sublethal CCl_4_ (carbon tetrachloride) exposure previously shown to induce LGR5^+^ LPCs (Figure 3A). Individual *Timps* responded to liver injury in WT mice with different expression kinetics. Consistent with scRNA-Seq (Figure 2C), *Timp1* showed the most upregulation in both injury types. *Timp1* and *Timp2* peaked at 3 days after CCl_4_ whereas *Timp3* showed a steady induction over 9 days, suggesting early involvement of *Timp1* and *Timp2* but late involvement of *Timp3* in the repair processes following acute damage (Figure 3B).

After DDC, mouse survival (data not shown) and hepatic damage assessed through serum analyses (serum alanine transaminase [ALT], icterus; Supplemental Figure 6, A and B) showed no difference in liver regeneration capacity between our mouse cohorts. Levels of TGF-β, TNF-α, and HGF were comparable after DDC injury (Supplemental Figure 5, D–F). Periportal DR is a clinical feature of chronic hepatic injury that correlates with progenitor expansion (40, 41) and establishment of hepatobiliary junctions during remodeling (42). WT livers presented CK19^+^ bile duct cells around portal veins at 2 weeks and bridging portal collagen deposition, extensive DR, and proliferative biliary cells at 4 weeks. *T1^−⁄−^T3^−⁄−^* livers showed a greater density of CK19 positivity at 2 weeks but had fewer CK19^+^ cells and lower biliary cell proliferation and collagen deposition at 4 weeks, all pointing to reduced DR (Figure 3C and Supplemental Figure 6, D and E). Biphenotypic SOX9^+^HNF4α^+^ hepatocytes, capable of clonogenicity and transdifferentiation into cholangiocytes (43), visualized by immunofluorescence were also less abundant in *T1^−⁄−^T3^−⁄−^* livers (Figure 3C). Flow cytometry enumeration of the MIC1-1C3^+^CD133^+^CD26^–^ biliary cell population (44), known to express SOX9 and possess progenitor function (45), although in comparable numbers at 2 weeks, was also drastically reduced in the *T1^−⁄−^T3^−⁄−^* cohort at 4 weeks (Figure 3D and Supplemental Figure 6C). Tumor-associated calcium signal transducer 2 (*Tacstd2*; *Trop2*), an LPC marker expressed under the DDC regimen (40, 41), was significantly reduced in *T1^−⁄−^T3^−⁄−^* liver tissue at 4 weeks (Figure 3E). Laminin, important for LPC expansion (46), was histologically quantified, and no difference was noted (Supplemental Figure 6E). Notch activation (target genes *Hey1* and *Hey2*), required for LPC differentiation (47), as well as YAP (target gene *Ccn1*), required for ductular response in the DDC model (30), was lower in *T1^−⁄−^T3^−⁄−^* livers at 2 weeks after DDC (Supplemental Figure 5A and Supplemental Figure 6F) but comparable at 4 weeks, suggesting altered kinetics of pathway activation during the ongoing damage and repair process in mice lacking *Timp1* and *Timp3*.

After CCl_4_, mouse survival was comparable (Supplemental Figure 7A), and characterization of WT and *T1^−⁄−^T3^−⁄−^* liver tissue at day 3 showed similar levels of pericentral necrosis, desmin-positive mesenchymal cell migration within the necrotic area, and HNF4α^+^Ki67^+^ proliferative zone 1 and 2 hepatocytes (Figure 3F and Supplemental Figure 7B). Although some *T1^−⁄−^T3^−⁄−^* mice showed a significantly higher level of ALT at day 3 after CCl_4_ (Supplemental Figure 7C), both genotypes showed resolution of pericentral damage with few residual proliferative cells and similar levels of total bilirubin and ALP at day 6 after CCl_4_ (Supplemental Figure 7D). Following this acute liver injury, TGF-β, TNF-α, and HGF were found transiently elevated in *T1^−⁄−^T3^−⁄−^* (TGF-β and TNF-α at 6 days and HGF at 9 days; Supplemental Figure 5, D–F), while the YAP pathway was not modulated (Supplemental Figure 5A). Flow cytometry enumeration revealed a reduction in the absolute number of MIC1-1C3^+^CD133^+^CD26^–^ cells in *T1^−⁄−^T3^−⁄−^* liver (Figure 3G). While the proportion of LPCs increased at day 6 after CCl_4_ in WT, this population remained lower in *T1^−⁄−^T3^−⁄−^* (Figure 3G and Supplemental Figure 7E). Among the LPC markers (40, 48, 49) *Sox9*, *Epcam*, *Trop2*, and Forkhead box L1 (*Foxl1*) measured by RT-PCR, *Epcam* showed consistent lower expression in these livers (Supplemental Figure 7F). Moreover, unchallenged 1-year-old *T1^−⁄−^T3^−⁄−^* mice had a smaller MIC1-1C3^+^CD133^+^CD26^–^ LPC population than aged WT mice (Supplemental Figure 3F). Overall, WT livers maintain and/or expand their LPC pool, but the combined lack of TIMP1 and TIMP3 results in LPC exhaustion following liver insults.

### Liver colony-forming capacity assays indicate a progenitor deficit in T1^−⁄−^T3^−⁄−^ mice.

We next set out to enumerate liver progenitors using a 2D colony-forming cell (CFC) assay (Figure 3, A and H). Total non-parenchymal cell fraction (100,000 cells plated), obtained after removal of hepatocytes, formed 2 types of colonies that were scored as holoclone and meroclone based on colony shape, cell morphology, and differentiation features, as described for epidermal colonies (50). Meroclone colonies comprised enlarged cells with high cytoplasm/nucleus ratio, which appeared lighter after Giemsa staining (Supplemental Figure 7G); these cells exhibited epithelial-like cell morphology after 12 days in culture (Supplemental Figure 7G), and some scattered cells were positive for the specification marker hepatocyte nuclear factor 4α (HNF4α). Holoclone colonies were generally smaller with a spindle contour, composed of tight small dark cells with a low cytoplasm/nucleus ratio (Supplemental Figure 7G). Holoclone was the only colony type found in cultures after serial total cell passaging of WT and *Timp*-deficient cells, and thus likely contained cells with self-renewal capacity. We found a high proportion of differentiated meroclone colonies and a lower number of holoclone colonies at 3 days after CCl_4_ in *T1^−⁄−^T3^−⁄−^* livers compared with both WT and its untreated control, indicating depletion of LPCs at the onset of tissue damage (Figure 3H). Total CFCs increased on day 6 in WT but remained low in the knockout group. As such, the proportion of holoclones, bearing self-renewal capacity, increased 3.3-fold (22% ± 13% to 73% ± 2%; *P* = 0.0147) in WT but only 1.7-fold in *T1^−⁄−^T3^−⁄−^* (34% ± 5% to 58% ± 16%; *P* = 0.5858) compared with their respective untreated controls. Thus, CFC enumeration, a well-accepted surrogate for measuring tissue stem/progenitor cells, indicated a smaller LPC pool in mice lacking TIMP1 and TIMP3 after liver injury.

### T1^−⁄−^T3^−⁄−^ bile duct fragments produce differentiated 3D organoids.

**Figure 4:**
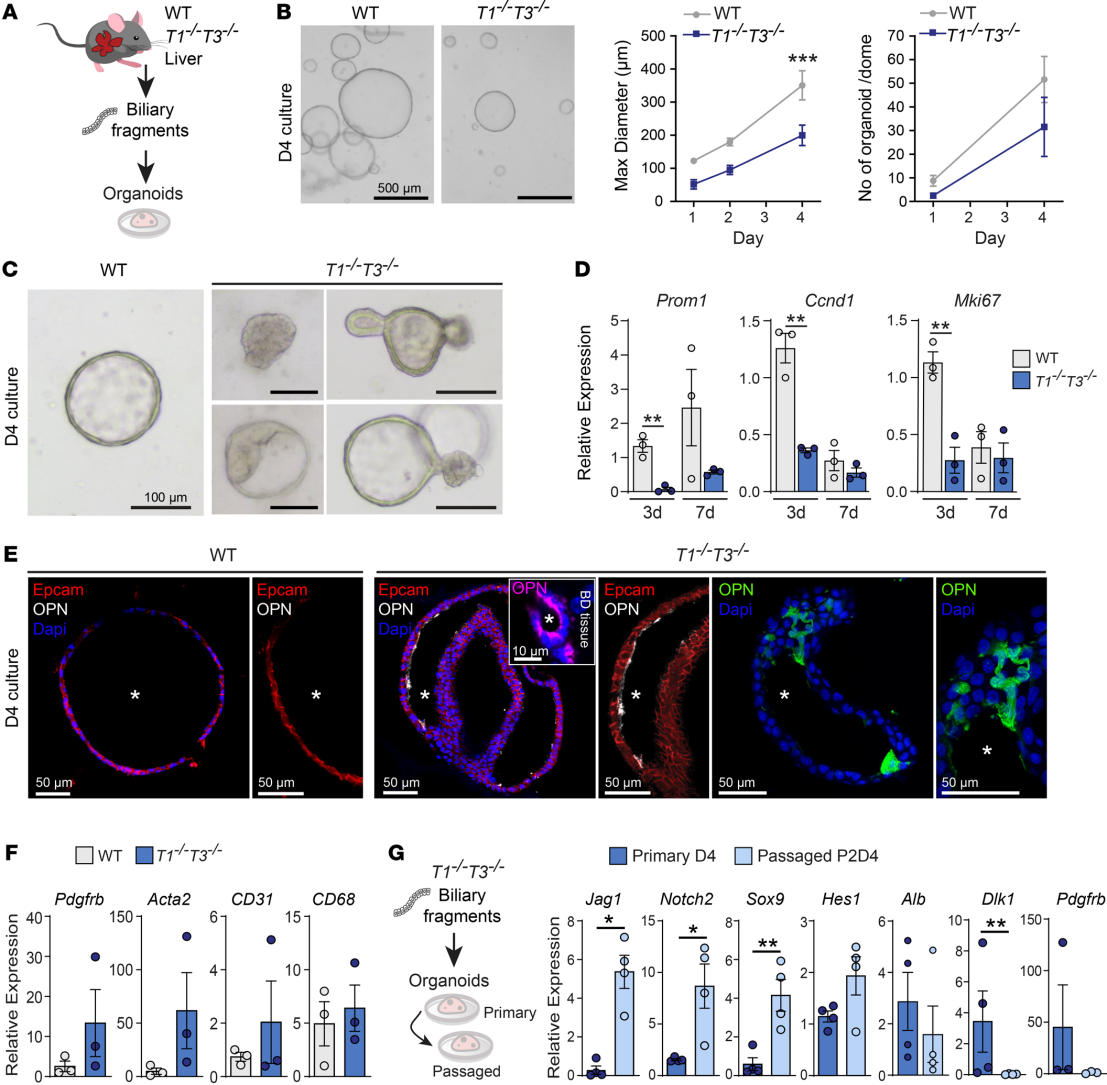
*Timp1* and *Timp3* loss impairs liver organoid formation. (**A**) Schematic of biliary fragment–derived liver organoid cultures. (**B**) Bright-field images of organoids, maximum diameter, and number of organoids per Matrigel dome during 4 days of culture; *n* = 5 livers. Two-way ANOVA with Šidák’s multiple-comparison test. (**C**) Images depict aberrations observed in *T1^−⁄−^T3^−⁄−^* organoids. Top middle, cell aggregates and absence of lumen; bottom middle, thickening of outer cell layer; top and bottom right, asymmetric growth. (**D**) Gene expression for progenitor marker *Prom1* and proliferation (*Ccnd1*, *Mki67*) during organoid growth; *n* = 3 livers per group. Two-tailed Student’s *t* test. (**E**) Immunofluorescence for markers of biliary cells (Epcam) and mature cholangiocytes (osteopontin, OPN) at day 4 culture of WT and *T1^−⁄−^T3^−⁄−^* organoids. Image inset in *T1^−⁄−^T3^−⁄−^* organoids panel shows OPN apical expression in bile duct (BD) from liver tissue; asterisk highlights lumen. (**F**) RT-PCR for cell population markers; *n* = 3 livers. Two-tailed Student’s *t* test. (**G**) Schematic of biliary fragment–derived primary organoids and their subsequent passages. Gene expression profiling of primary and passaged organoids; *n* ≥ 3 livers. Ratio paired *t* test, *P < 0.05, **P < 0.01, ***P < 0.001.

3D organoids recapitulate tissue biology and are currently the preferred system to study progenitor cell behavior. Our analyses thus far had exposed aberrations in the biliary cell compartment of *T1^−⁄−^T3^−⁄−^* mice; therefore we established biliary fragment–derived organoids (51). Specifically, biliary fragments extracted from the whole liver were cultured to capture the entire biliary LPC population (Figure 4A). Primary *T1^−⁄−^T3^−⁄−^* organoids were less numerous and smaller than WT (Figure 4B), developing at a slower rate and never reaching the maximum organoid size seen in WT. Notably, *T1^−⁄−^T3^−⁄−^* organoids generated from livers 6 days after CCl_4_ showed a profound delay in organoid formation and growth (Supplemental Figure 8A). In WT cultures, 4 times more organoids formed and reached a larger size compared with untreated livers (day 1; Figure 4B and Supplemental Figure 8A). Furthermore, multiple dense “filled” organoids remained at day 4 (Figure 4C) in *T1^−⁄−^T3^−⁄−^* cultures, a time point when the expected hollow cystic WT organoids had fully formed. Structural aberrations in these *Timp*-deficient organoids included thickening of the outer cell layer and asymmetric shape (Figure 4C). In WT organoids, the progenitor marker *Prom1* increased and proliferation (*Ccnd1* and *Mki67*) decreased as they matured from day 3 to day 7 (Figure 4D). *T1^−⁄−^T3^−⁄−^* organoids had lower proliferation and *Prom1* levels, prompting us to investigate their differentiation status (Supplemental Figure 8B); they expressed multiple mature cholangiocyte markers *Krt19*, *Cftr*, and *Spp1* along with altered *Adam* and Notch ligand/receptor profiles. Immunofluorescence revealed formation of osteopontin-positive (OPN^+^) ductal structures, and polarized OPN protein in “filled” *T1^−⁄−^T3^−⁄−^* organoid sections resembled the natural OPN expression in biliary duct in vivo (Figure 4E, inset). These data collectively support the reduction of biliary LPC content in *T1^−⁄−^T3^−⁄−^* livers and demonstrate their increased propensity to differentiate in vitro.

We had observed that *Dlk1* expression was mostly restricted to mesenchymal cells in scRNA-Seq (Figure 2C) yet was clearly detected in organoid cultures of both genotypes, implying the presence of other cell populations in organoid cultures (Supplemental Figure 8B). RT-PCR analysis for stromal cell type markers that typically make up the tissue niches confirmed the presence of endothelial cells (*CD31*), macrophages (*CD68*), mesenchymal cells (*Pdgfrb*), and VSMCs (*Acta2*) in the fragment-derived cultures (Figure 4F). Markers for mesenchymal cells were generally higher in *T1^−⁄−^T3^−⁄−^* cultures. With subsequent passaging, expression of the mesenchymal cell markers *Pdgfrb* and *Dlk1* faded (Figure 4G and Supplemental Figure 8C). Organoids showed enrichment of genes known to be highly expressed in biliary cells (*Jag1*, *Notch2*, *Sox9*, and *Hes1*; Figure 2C) and continued to express albumin, suggesting maintenance of cell plasticity. Additionally, *Timp1*, *Timp2*, and *Timp3* were found in fragment-derived WT organoid cultures as well as in passaged organoids, indicating the ability of LPCs to produce these TIMPs. In contrast to the biliary fragment–derived organoid cultures that contained niche cells, organoids were next generated from the FACS-purified MIC1-1C3^+^CD133^+^CD26^–^ progenitor population, which was devoid of other cell types (Supplemental Figure 8D). When the same number of WT and *T1^−⁄−^T3^−⁄−^* MIC1-1C3^+^CD133^+^CD26^–^ cells were plated, no difference was observed in number, size, or morphology of organoids. These data show that the presence of stromal cells was key to the regulation of LPC fate and the underlying distinctions observed in *T1^−⁄−^T3^−⁄−^* organoid differentiation.

### ADAMs and DLK1 are coexpressed by periportal mesenchymal cells.

**Figure 5:**
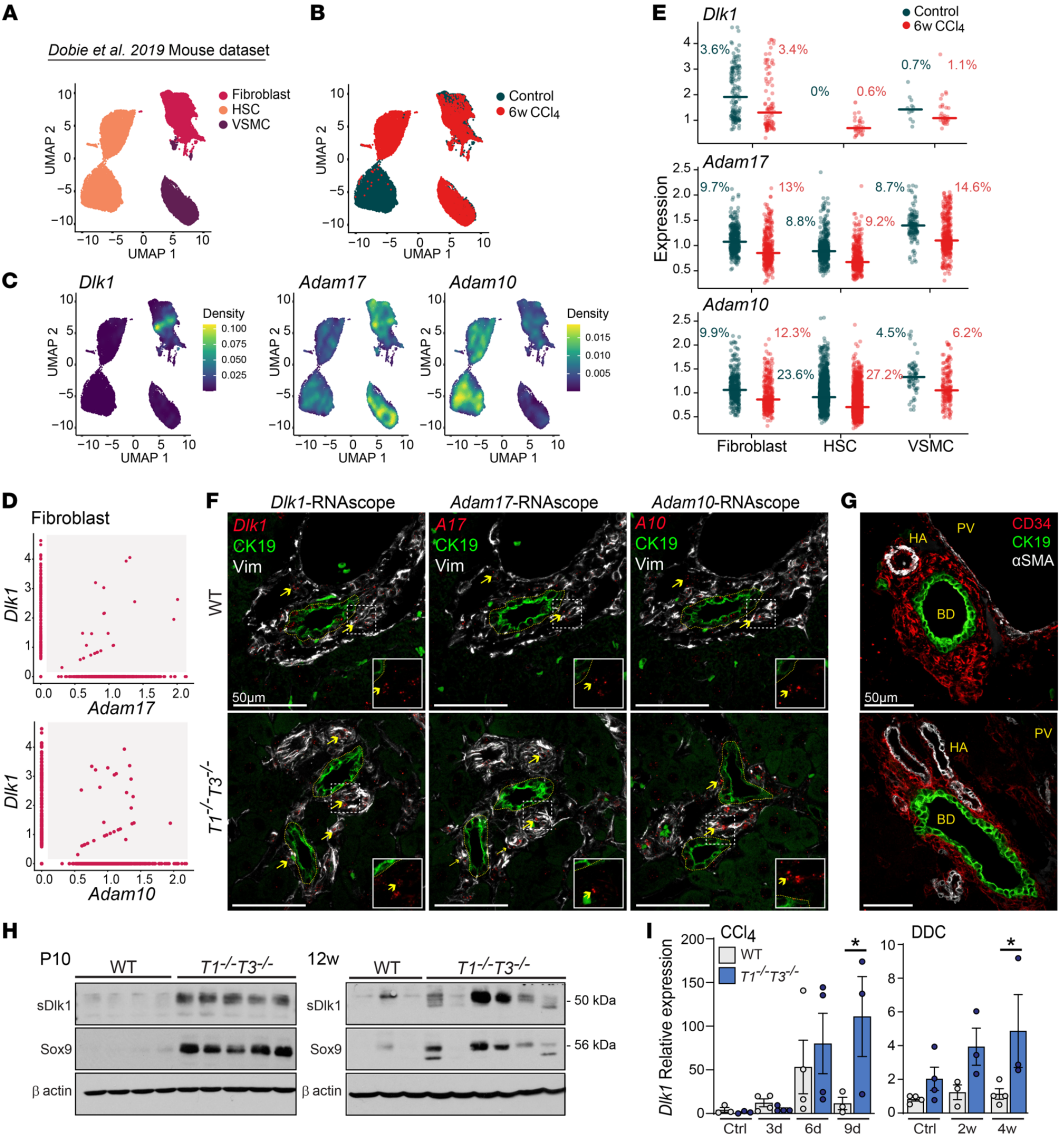
ADAMs and DLK1 are coexpressed in periportal mesenchymal cells. (**A**) UMAP visualization of isolated hepatic mesenchymal cells, based on 23,689 PDGFRβ+ single-cell transcriptomes pooled from control mice (12,587 cells) and after 6 weeks CCl_4_ treatment (11,102 cells) from the Dobie et al. 2019 mouse dataset (52) (HSC, hepatic stellate cell; VSMC, vascular smooth muscle cell). (**B**) UMAP visualization of control and CCl_4_-treated cells. (**C**) Density plots showing expression of *Dlk1*, *Adam10*, and *Adam17* in liver mesenchymal cell populations described in **A**. (**D**) Correlation plots representing fibroblasts expressing *Dlk1*, *Adam17*, and *Adam10*. The gray area contains cells coexpressing *Dlk1*/*Adam17* and *Dlk1*/*Adam10*. (**E**) Scatterplots showing expression level of *Dlk1*, *Adam10*, and *Adam17* in fibroblasts, HSCs, and VSMCs. Percentages indicate the number of cells expressing the gene of interest among the total cell population. (**F**) Representative RNAscope and immunofluorescence images of biliary ducts from WT and *T1^−⁄−^T3^−⁄−^* untreated adult livers (*n* = 3). CK19^+^ biliary cells forming ducts are delineated with yellow dashed lines. Yellow arrows point to *Dlk1*, *Adam10*, or *Adam17* RNAscope positive staining in vimentin-positive (Vim) mesenchymal cells. (**G**) Immunofluorescence image of WT and *T1^−⁄−^T3^−⁄−^* portal triad showing CK19^+^ cholangiocyte, CD34^+^ fibroblast, and αSMA^+^ VSMC (PV, portal vein; HA, hepatic artery; BD, bile duct). (**H**) Western blot for cleaved DLK1 (sDLK1, 50 kDa) and SOX9 in postnatal and adult livers; each lane represents 1 animal. (**I**) Expression of *Dlk1* in liver tissue following CCl_4_ or DDC injury (*n* ≥ 3). One-way ANOVA with Šidák’s multiple-comparison test, *P < 0.05.

To better decipher the cellular landscape of ADAM and DLK1 expression in the portal microenvironment, we first interrogated an independent published scRNA-Seq dataset derived from purified liver mesenchymal cells from control mice and post-CCl_4_ chronic injury (Dobie et al., 2019, ref. 52) and found the expected major clusters: portal fibroblasts, HSCs, and VSMCs (Figure 5, A and B). Density plots confirmed *Dlk1* expression in fibroblasts (Figure 5C), with subsets of cells coexpressing *Adam17* (2.4%) or *Adam10* (3.2%) along with *Dlk1* (Figure 5D). Although *Adam10* and *Adam17* were present in all populations, *Adam17* was high in fibroblasts and VSMCs while *Adam10* was high in HSCs (Figure 5E). Scatterplots revealed that *Dlk1* could also be detected in VSMCs. After chronic CCl_4_-induced hepatic damage, the proportion of cells expressing *Adams* increased, and *Dlk1* showed an induction in HSCs. ADAM17, a sheddase specifically inhibited by TIMP3, is a transmembrane protease that converts membrane-bound DLK1 to its soluble form (sDLK1), enabling SOX9 induction in preadipocytes (15, 53). Therefore, we next visualized the baseline spatial localization of *Dlk1*, *Adam10*, and *Adam17* in the liver using a combination of in situ hybridization and immunofluorescence on serial tissue sections (RNAscope; Figure 5F). Vimentin-positive mesenchymal cells in the vicinity of bile ducts coexpressed *Dlk1*, *Adam10*, and *Adam17*, and these cells were identified as fibroblasts (CD34^+^) and VSMCs (αSMA^+^) by independent immunofluorescence (Figure 5G). Comparison of WT and *T1^−⁄−^T3^−⁄−^* livers highlighted high numbers of *Adam10/17-*expressing VSMCs in very close proximity to bile ducts, underscoring the altered portal niches in *Timp*-deficient liver that in turn could affect sDLK1 bioavailability. Western blot analysis of bulk tissue showed higher levels of sDLK1 (50 kDa), which consistently correlated with SOX9, in postnatal P10 as well as unchallenged adult *T1^−⁄−^T3^−⁄−^* livers (Figure 5H). Additionally, both acute and chronic liver injuries induced higher *Dlk1* expression in *T1^−⁄−^T3^−⁄−^* liver (Figure 5I). Altogether, periportal mesenchymal cells are normally equipped to release sDLK1 in the LPC niche, whereas the aberrant *T1^−⁄−^T3^−⁄−^* portal milieu is conducive to excessive local sDLK1 delivery.

### sDLK1 promotes biliary progenitor differentiation.

**Figure 6:**
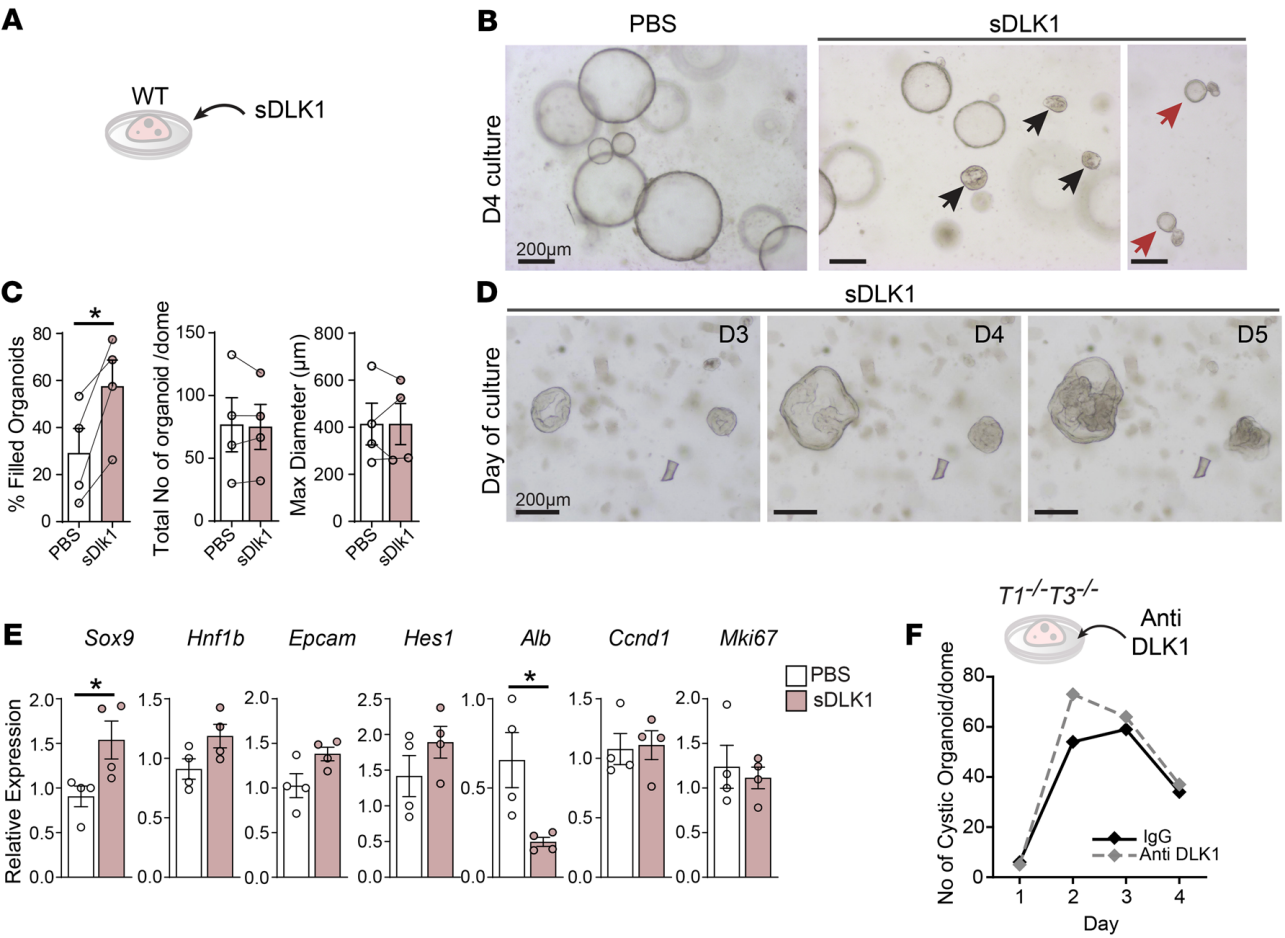
Soluble DLK1 induces *Sox9* expression and biliary differentiation of organoids. (**A**) WT organoids treated with recombinant soluble DLK1 (sDLK1; 400 ng/mL). (**B**) Bright-field images of PBS- or sDLK1-treated organoids; black arrows point to “filled” organoids, and red arrows show asymmetric growth. (**C**) Scatterplots show percentage of organoids with “filled” phenotype, total organoid number per dome, and their maximum size after 4-day treatment; *n* = 4 livers. Paired *t* test, *P < 0.05. (**D**) Time course of “filled” organoid growth in the presence of sDLK1. (**E**) Expression of canonical Notch target gene (*Hes1*) and markers of cholangiocytes (*Sox9*, *Hnf1b*, *Epcam*), hepatocytes (*Alb*), and proliferation (*Ccnd1*, *Mki67*) in organoids after sDLK1 treatment; *n* = 4 livers per group. Two-tailed Student’s *t* test, *P < 0.05. (**F**) Kinetics of *T1^−⁄−^T3^−⁄−^* cystic organoid formation in the presence of IgG control or anti-DLK1 antibody (2 ng/μL).

Increased biliary differentiation and sDLK1 bioavailability with high SOX9 levels were striking features of *T1^−⁄−^T3^−⁄−^* liver. We tested whether sDLK1 directly influences LPC fate in WT organoids (Figure 6A); addition of recombinant sDLK1 resulted in morphological defects including a thickened outer layer and asymmetric growth (Figure 6B) mimicking features of *T1^−⁄−^T3^−⁄−^* organoids (Figure 4C). Total number and maximum size of organoids remained unaltered, but a greater proportion of organoids were filled with ductal structures after sDLK1 treatment (Figure 6C). Tracking individual organoids revealed the constant accumulation of such structures until their collapse, without having reached their full expansion potential (Figure 6D). Quantitative RT-PCR analysis of a panel of genes showed a significant increase in the biliary marker *Sox9* with a concomitant downregulation of the hepatocyte marker albumin (*Alb*) after 3 days of culture (Figure 6E). Finally, we examined whether *T1^−⁄−^T3^−⁄−^* organoid growth could be improved by neutralizing of sDLK1 using an anti-DLK1 antibody specific to the DLK1 ectodomain. The number of clear cystic organoids increased within 3 days (Figure 6F), indicating that blocking sDLK1 rescues normal organoid formation and LPC expansion. Thus, sDLK1 is an important inducer of *Sox9* and a trigger for cholangiocyte specification, supporting its critical function in the LPC niche.

### ADAM inhibitors prolong LPC expansion and organoid survival.

**Figure 7:**
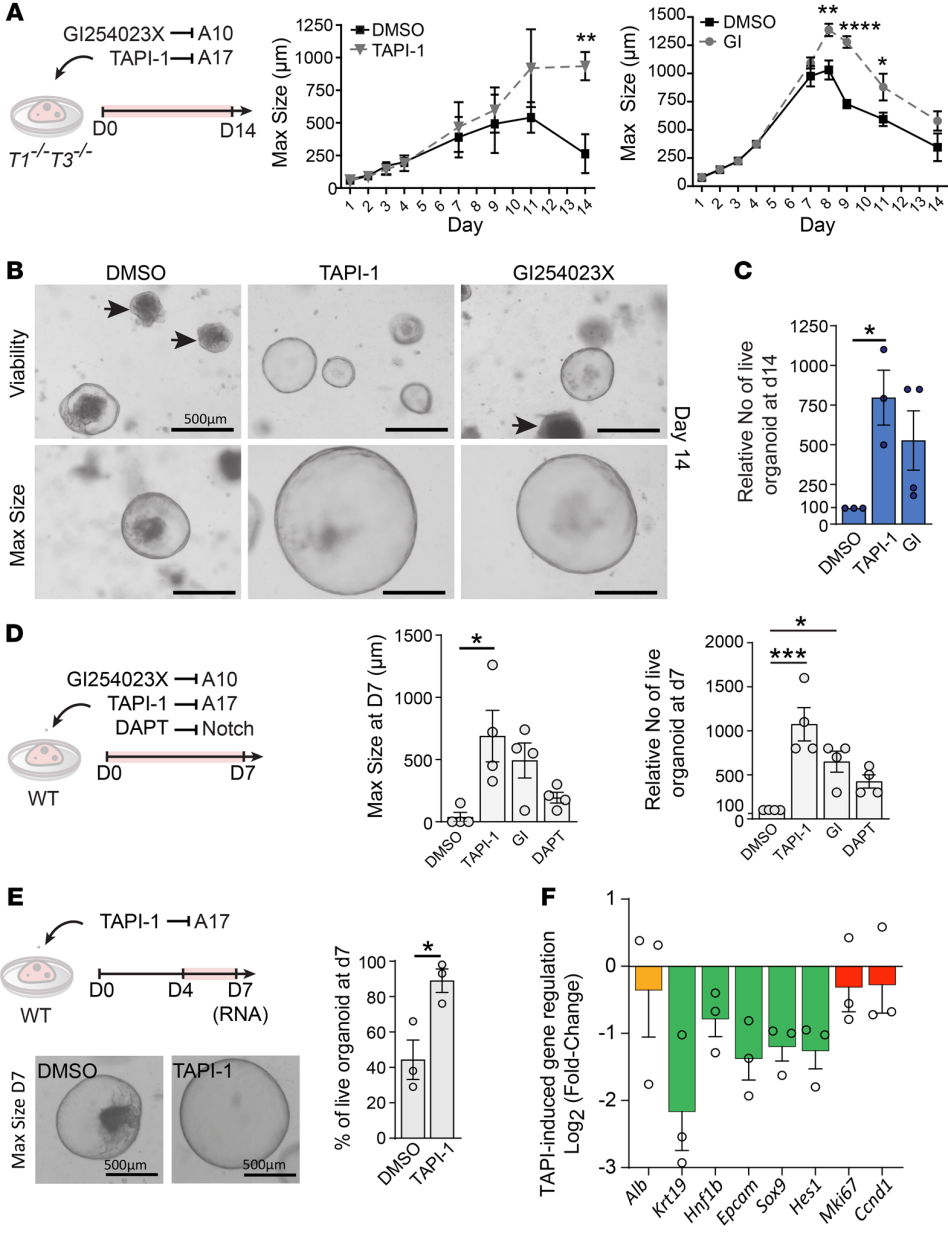
ADAM inhibitors prolong LPC expansion and organoid survival. (**A**) *T1^−⁄−^T3^−⁄−^* biliary organoids were treated with ADAM inhibitors for 14 days. Time course of organoid growth using maximum diameter in the presence of the ADAM inhibitors GI254023X (GI; *n* = 4 livers) and TAPI-1 (*n* = 3 livers). Two-way ANOVA with Šidák’s multiple-comparison test. (**B**) Bright-field images of organoid cultures in the presence of TAPI-1 and GI254023X, showing collapsed organoids as black cell aggregates (arrows) and size of the largest organoid at day 14. (**C**) Relative number of *T1^−⁄−^T3^−⁄−^* live organoids in the presence of ADAM inhibitors compared with DMSO (100%). One-way ANOVA with Bonferroni’s multiple-comparison test. (**D**) WT organoids treated with ADAM and Notch inhibitors for 7 days. Maximum organoid size and relative number of WT live organoids obtained in the presence of TAPI-1, GI254023X, and DAPT compared with DMSO; *n* = 4 livers. One-way ANOVA with Bonferroni’s multiple-comparison test. (**E**) WT biliary organoids treated with TAPI-1 from day 4 to day 7. Bright-field images of the largest organoids and percentage of live organoids at day 7; *n* = 3 livers. Two-tailed paired *t* test. (**F**) TAPI-1–induced gene expression regulation of *Alb* (hepatocyte); *Krt19*, *Hnf1b*, and *Epcam* (cholangiocyte); *Hes1* and *Sox9* (biliary differentiation); and *Mki67* and *Ccnd1* (proliferation) in day 7 organoids, expressed as log_2_ of the fold change (TAPI-1/DMSO). *P < 0.05, **P < 0.01, ***P < 0.001, ****P < 0.0001.

Since Notch activation and sDLK1 release require sheddase activity, we measured the effects of ADAM small-molecule inhibitors on organoids (Figure 7A). Addition of GI254023X, which blocks ADAM10, or TAPI-1, which blocks ADAM17, to *T1^−⁄−^T3^−⁄−^* cultures for 14 days showed their potent effect on LPC expansion and organoid survival over this extended period. Specifically, the vast majority of DMSO-treated organoids began collapsing by day 7, whereas both ADAM inhibitors allowed organoids to sustain growth and reach larger diameters from day 8 to day 14 (Figure 7B). A higher percentage of live organoids was observed at day 14, with TAPI-1 exhibiting the strongest pro-survival effect (Figure 7C). Thus, restraining of ADAM10 or ADAM17 activity is crucial for adequate LPC expansion and survival. In the WT organoid setting, we also tested the effect of DAPT, known to abrogate Notch activity by blocking γ-secretase (Figure 7D). Notch inhibition also promoted LPC survival. The pro-survival effect of TAPI-1 was superior to that of GI254023X or DAPT. Since inhibiting ADAM10 or ADAM17 did not affect organoid initiation but affected solely the later stage of organoid growth, we next added TAPI-1 from day 4 to day 7 (Figure 7E). Around 50% of WT organoids collapsed in control conditions by day 7, yet nearly all organoids remained healthy in the presence of TAPI-1. Gene expression profiling confirmed downregulation of biliary markers (*Krt19* and *Epcam*) and Notch target genes (*Hes1*, *Sox9*) without influencing proliferation (*Ccnd1* and *Mki67*) (Figure 7F). Therefore, control over ADAM activity is crucial to preserve LPC expansion and restrict cholangiocyte specification.

### sDLK1 induces SOX9 in human hepatic organoids.

**Figure 8:**
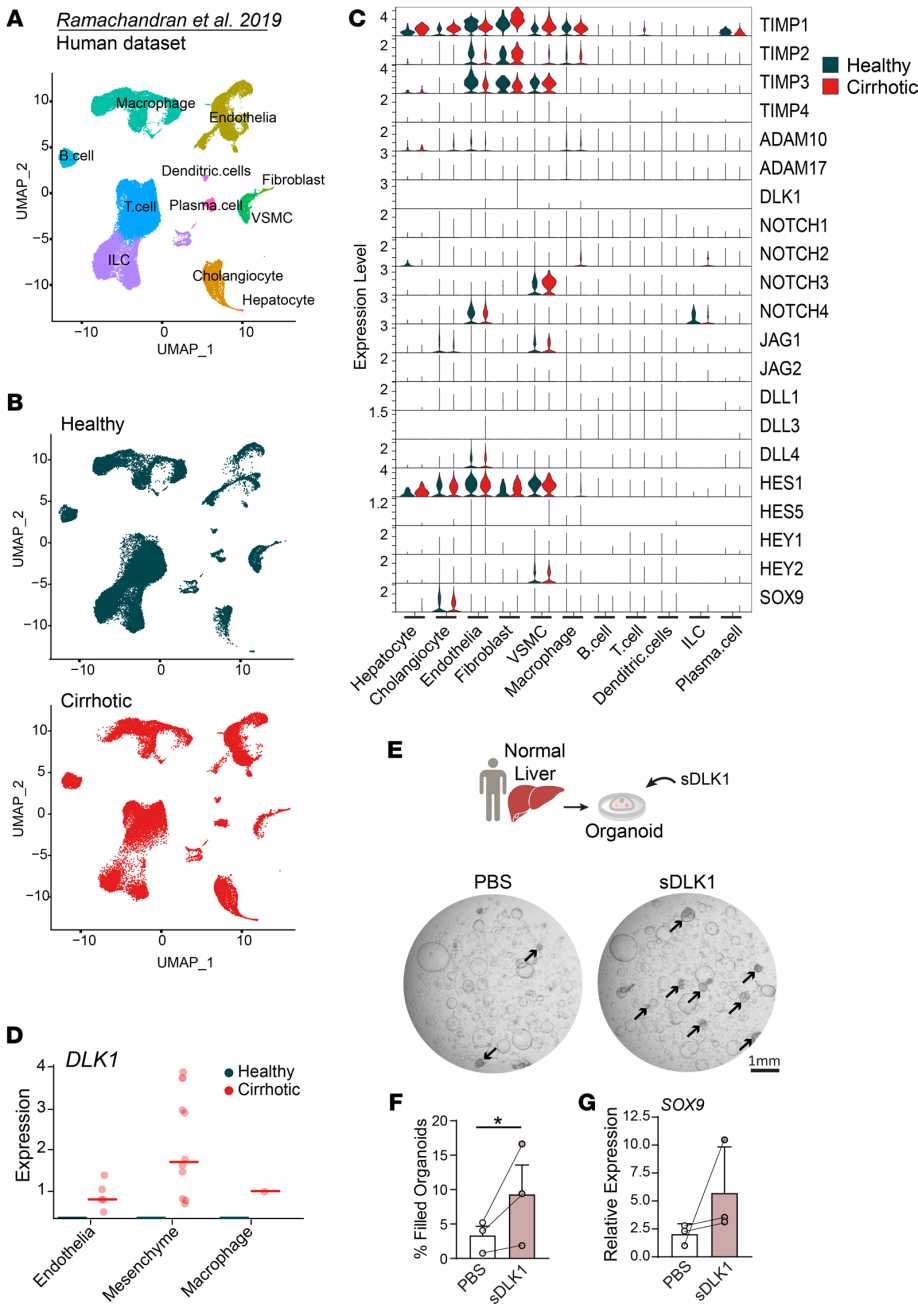
sDLK1 induces SOX9 and a filled morphology in human liver organoids. (**A**) UMAP visualization of isolated hepatic cells, based on 66,135 single-cell transcriptomes pooled from 5 healthy and 5 cirrhotic human livers from the Ramachandran et al. 2019 dataset (54). (**B**) UMAP obtained for each condition. (**C**) Violin plots showing the expression of TIMP, ADAM, NOTCH ligands, receptor, and target genes in the hepatic cell populations defined in **A** for healthy and cirrhotic patients. (**D**) Scatterplots showing mean expression of *DLK1* in human hepatic cells in healthy and cirrhotic conditions. (**E**) Human liver organoids treated with PBS (control) or sDLK1 recombinant protein (400 ng/mL). *Z*-stacking of bright-field images of entire Matrigel dome; arrows show filled organoids. (**F**) Percentage of filled organoids after sDLK1 treatment. (**G**) RT-PCR for *SOX9* expression in treated organoids; *n* = 3 patients (2 males, 1 female). Two-tailed ratio paired *t* test analysis, *P < 0.05.

Finally, we interrogated the human liver scRNA-Seq dataset from Ramachandran et al., 2019 (54), which includes healthy and cirrhotic liver states (Figure 8, A and B). We identified comparable expression patterns of the TIMP/ADAM/NOTCH/DLK1 axis components between humans (Figure 8C) and mice as detailed above (Figure 2C). Among noted differences, *TIMP1* was constitutively expressed in healthy human liver, and not restricted to mesenchymal cell populations. With respect to *DLK1*, it was mainly detected in fibroblasts of the cirrhotic liver (Figure 8D). We tested whether sDLK1 could serve a similar function of promoting LPC differentiation in the human cell setting. We derived normal hepatic organoids from 3 patient surgical resection specimens, and these were cultured in the presence of human recombinant sDLK1 (Figure 8E). Although no differences were observed in the number and size of organoids formed, a significantly higher number of organoids was noted to be dense and filled (Figure 8F). Importantly, this was accompanied by an induction of *SOX9* expression in sDLK1-treated human organoids (Figure 8G). These data document the direct effect of sDLK1 on human liver progenitors, setting them on a path to cholangiocyte differentiation.

## Discussion

Understanding tissue stem cell programs is imperative to overcome barriers in cell therapy for regenerative medicine. In the liver, progenitor cells participate in regeneration after severe injury, and systems underlying their regulation are of wide interest (1, 2). Through a multitiered approach with in vivo analyses of murine livers and gold standard in vitro systems designed to study both murine and human liver progenitor biology, we have now uncovered the combined function of TIMP1 and TIMP3 in biliary development and LPC maintenance. LPCs are skewed toward exhaustive cholangiocyte differentiation in the absence of these TIMPs and are unable to expand after liver injury. We delineate mechanisms underlying this biology, specifically underscoring the role of the periportal niche, the molecular interplay of TIMPs and ADAMs in Notch activation, and soluble DLK1 bioavailability as a critical effector of cholangiocyte specification in the liver. The metalloproteinase-inhibitor system regulates multiple aspects of tissue homeostasis and liver biology, including hepatocyte cell cycle kinetics, death, and senescence (13, 33, 34, 55, 56). Complementing the previously shown requirement of combinations of TIMPs in regulating mammary stem cells, chondrocyte differentiation, and the bone marrow hematopoietic niche (22, 57–59), this study broadens the foundational role of this major protease system in maintaining adult tissue stem cells and regulating progenitor cell fate.

Privileged stem cell niches are known to maintain the characteristics of stem cells, balancing their quiescence, activation, and subsequent differentiation. In patients with chronic hepatitis and rodent models of liver injury, LPCs colocalize with laminin, endothelial cells, myofibroblasts, and macrophages (60). Interactions among LPCs, stromal cells, extracellular matrix, growth factors, and cytokines are injury dependent and determine progenitor cell behavior (47). Our interrogation of mouse and human scRNA-Seq data mapped this cellular network, pinpointing critical paracrine and juxtacrine signals within the LPC niche. Specifically, within the ADAM and Notch ligand/receptor landscape we verified that portal mesenchymal cells coexpress ADAM17 and DLK1 in situ and are equipped to release sDLK and direct LPC differentiation in a paracrine manner. The combined loss of TIMP1 and TIMP3, the natural inhibitors of ADAM10 and ADAM17, culminated in abundant cleaved NICD and DLK1 in liver homogenates, reflective of unchecked ADAM shedding activity. Additionally, our bioinformatic interrogation indicated a juxtacrine interaction with mesenchymal cells expressing JAG1 that can activate Notch signaling in LPCs, which possess both Notch receptor 2 and ADAM10. Finally, given that periportal mesenchymal cells have recently been reported to regulate mature cholangiocyte proliferation through secreted factors and Notch signaling (61), these cells have the potential to influence both mature cells and LPCs. Thus, we have exposed key components of the TIMP/ADAM/NOTCH/DLK1 axis that operate in both a paracrine and a juxtacrine manner, to regulate LPC fate within the hepatic periportal niche.

In our organoid cultures, which recapitulate many aspects of the complex structure and function of in vivo tissue, lower progenitor activity in *T1^−⁄−^T3^−⁄−^* livers was due to alterations of the LPC niche rather than a cell-autonomous feature. Specifically, owing to the presence of stromal cells, *T1^−⁄−^T3^−⁄−^* biliary fragments produced differentiated organoids capable of only limited growth, while the FACS-purified MIC1-1C3^+^CD133^+^CD26^−^ LPC fraction generated organoids with morphology and growth kinetics comparable to WT. This supports the role of stroma in LPC fate determination. TIMP1 induced in mesenchymal cells and TIMP3 constitutively expressed by biliary, mesenchymal, and endothelial cells are secreted endogenous inhibitors ideally positioned to modulate the availability of local signals in the LPC niche. TIMP3 is the only TIMP to bind the extracellular matrix, and it can stabilize the cell microenvironment, serving to sustain progenitors within the hepatic niche. TIMP3 is implicated in the regulation of other tissue stem cells as it suppresses myogenic differentiation in the muscle (62) and maintains the undifferentiated state of murine embryonic neural stem cells in the brain (63). Elevated sDLK1 correlated with high Sox9 protein levels in *T1^−⁄−^T3^−⁄−^* hepatic tissue. Exogenous ADAM17 inhibition reduced *Sox9* expression and cholangiocyte differentiation and substantially prolonged liver organoid growth. ADAM10 or Notch inhibition also increased organoid survival, although this effect was inferior to that of ADAM17 inhibition, shedding new light on the ADAM17-sDLK1 pair in LPC differentiation. Moreover, experimental modulation through exogenous recombinant sDLK1 and neutralizing anti-DLK1 antibody of WT and TIMP-deficient organoids, respectively, confirmed sDLK1 as a powerful cholangiocyte specification factor acting through *Sox9* induction.

*Sox9* is a recognized Notch target gene, and sDLK1 has also been shown to activate *Sox9* in preadipocytes via fibronectin binding and MAPK/ERK activation, independently of Notch (53); higher ERK activation was notable in adult *T1^−⁄−^T3^−⁄−^* liver. Cell signaling through DLK1 is understudied, although its importance in tissue stem cells is emerging (16). For instance, sDLK1 secreted by niche astrocytes regulates neural stem cell self-renewal through interaction with its membrane-bound form (64), and comparison of fetal hepatic stem cells expressing both membrane-bound and soluble forms of DLK1 versus sDLK1 alone has shown that the latter enhances cholangiocyte commitment in vitro (65). Herein, sDLK1 triggered *Sox9* upregulation and cholangiocyte differentiation in both mouse and patient-derived hepatic organoids, attesting to the conserved powerful function of sDLK1.

Elucidating the cellular and molecular signals modulating LPC fate has the potential to pave the way for future liver disease therapy. Combined TIMP-knockout models provide valuable tools to deconvolute the microenvironmental cues in a physiological manner, exposing critical proteolytic events responsible for cell fate decisions. Our findings broaden the role of the metalloproteinase-inhibitor system in maintaining adult tissue stem cells and underscore the potential application of metalloproteinase inhibitors in generating an environment conducive to adult tissue progenitor cell expansion. Manipulating metalloproteinase activity may provide a means to influence biliary progenitor cells in vivo and in organoids ex vivo, opening new avenues for regenerative medicine approaches.

## Methods

### Sex as a biological variable

Our human study examined male and female patients, and similar findings were observed. Our mouse study examined male mice because sex disparity has been reported in liver disease; however, results were expected to be relevant regardless of biological sex based on the results of our human study.

### Patients

Normal adjacent liver tissue was obtained intraoperatively from 2 male patients (ages 29 and 45) and 1 female patient (age 55) undergoing surgical liver resection for intrahepatic cholangiocarcinoma at Toronto General Hospital (Toronto, Ontario, Canada).

### Mice

*Timp1^−⁄−^Timp3^−⁄−^* whole-body-knockout mice were generated by crossing of reported *Timp1^−⁄−^* and *Timp3^−⁄−^* mice in pure C57BL/6 backgrounds (66, 67), backcrossed at least 6 times. Mouse genotypes were confirmed by PCR of genomic DNA. The absence of specific *Timp* transcripts was confirmed by PCR of cDNA generated from tissues with the highest levels of transcription of *Timp1* (ovary) and *Timp3* (kidney). All experiments used male mice, except for embryos, of which both sexes were analyzed. WT C57BL/6J mice were purchased from The Jackson Laboratory (strain 000664). Mice were housed in a modified barrier, specific pathogen–free facility in sealed negative ventilation cages (Allentown; 22°C–24°C, 12-hour light/12-hour dark cycle, ad libitum food and water) with up to 5 mice per cage.

#### Genotyping.

PCR was performed on genomic DNA isolated from pups using genomic *Timp1* and *Timp3* primers listed in Supplemental Table 1. Three-primer PCR formed a larger band with the common/WT primers than with the common/neo primers, to distinguish between WT and mutant alleles. To confirm the disruption of gene expression in the whole-body knockouts, PCR was also performed on cDNA derived from RNA isolated from tissues with high levels of mRNA for *Timp1* (ovary) and *Timp3* (kidney). The PCR conditions (T_A_/number of cycles) were 60°C/40× for *Timp1* and *Timp3* primers and 62°C/60× for *Hprt*. The PCR products were electrophoresed on a 2% agarose gel alongside a 100 bp ladder (GeneDireX, DM001-R500).

### Liver injury models and tissue collection

*Timp1^−⁄−^Timp3^−⁄−^* and age-matched WT mice received a sublethal dose of CCl_4_/corn oil (v/v),1.9 ml/kg i.p. (Sigma-Aldrich) or corn oil vehicle to induce acute liver damage. Mice were euthanized at 3, 6, and 9 days after injection. For chronic hepatic damage, mice were fed a 5% fat chow diet with or without supplementation with 0.1% wt/wt DDC (3,5-diethoxy-16 carbonyl-1,4-dihydrocollidine; Sigma-Aldrich and Inotiv) for 2 or 4 weeks. Blood was collected from cardiac puncture for serum analysis, and icterus was detected photometrically on the Beckman AU480 Biochemistry Analyzer and scored by the Centre for Phenogenomics in Toronto, Ontario, Canada. For both models, the left liver lobe was tied off and cut in half, and one piece was snap-frozen for biochemistry and the other half fixed in 4% paraformaldehyde (PFA) and paraffin-embedded for histology. The remaining lobes were perfused with digestion buffers through the portal vein to obtain a single-cell suspension as described below.

### Liver organoid culture

#### Mouse organoids.

Biliary fragment–derived liver organoids were derived from *Timp1^−⁄−^Timp3^−⁄−^* and WT untreated or CCl_4_-treated mice, as described in schematics following the protocol and reagents from STEMCELL Technologies (DX21821). Briefly, biliary fragments, obtained after sequential digestion of the entire liver using a blend of collagenase IV and dispase (STEMCELL Technologies, 07909 and 07923, respectively), were collected on a strainer, split into 4 Matrigel domes (Corning, 356231), and cultured in Hepaticult growing medium (STEMCELL Technologies, 06030). For MIC1-1C3^+^CD133^+^CD26^−^ sorted cell culture, further dissociation of hepatic ducts into single cells was performed following STEMCELL Technologies procedure. Single cells were then labeled with primary antibody cocktail (Supplemental Table 2); DAPI was used as viability dye. MIC1-1C3^+^CD133^+^CD26^−^ cells were FACS sorted using FACSAria (BD), and 4,000 WT and *Timp1^−⁄−^Timp3^−⁄−^* cells were plated in 10 μL Matrigel dome in duplicate and cultured in Hepaticult growing medium supplemented with 10 μM Y-27632 (Millipore, SCM075) and 5 μM forskolin (Tocris, TB1099-RMU/10). For passaging organoids, 1 mL of cold TrypLE Express (Gibco) was added into wells containing domes and incubated at 37°C for 30 minutes to break Matrigel and dissociate organoids into single cells. Splitting ratio was determined according to organoid confluence. For organoid treatment, 2 domes were used as control and the other 2 as experimental condition. Inhibitors or recombinant DLK1 were incorporated into Matrigel dome at the time of seeding, and in growth medium at all times except in Figure 7E, where TAPI-1 was added in growth medium only from day 4 to day 7. All inhibitors were used at 20 μM: TAPI-1 (Peptide International, INH-3855-PI), GI254023X (Aobious, AOB3611), and DAPT (Sigma-Aldrich, D5942). Recombinant human DLK1 (Enzo Life Sciences, ALX-201.764.0010; 400 ng/mL) or anti-DLK1 antibody (R&D, AF8277; 1:100) were added in growth media for 4 days. Media were changed every 2 days.

#### Human organoids.

Normal liver surgical resections were processed to generate hepatic organoids following the protocol of Broutier et al. (68). After mincing and complete digestion of the tissue specimen, cell pellets were resuspended in 20 μL Matrigel domes. After expansion of patient-derived organoids through subsequent passages, cultures were treated (duplicate) with recombinant human DLK1 (Enzo Life Sciences, ALX-201.764.0010; 400 ng/mL) for 10–15 days. *Z*-stacked bright-field images of the domes were acquired using BioTek Cytation 5 Cell Imaging (Agilent) before addition of TRIzol reagent (Invitrogen) for RNA extraction.

### Single-cell preparation

Liver was perfused with 3 successive buffers — HEPES/EGTA (0.6 mM) for 6 minutes, HEPES for 3 minutes, and HEPES/CaCl_2_ (5 mM)/Liberase (12.5 μg/mL; Roche) for 4 minutes — through the portal vein at a flow rate of 3 mL/min to isolate liver cells. After mechanical dissociation of the liver in Leibovitz/5% FBS medium, the cells were then filtered (70 μm and 40 μm strainers) and left on ice. Incompletely dissociated tissue was recovered from the strainers and further digested using Collagenase D (2.5 mg/mL; Roche) for 30 minutes at 37°C. After 100 μg/mL DNase I (Sigma-Aldrich) treatment, the cells were filtered (40 μm) and combined with the first cell suspension. If liver tissue remained in the strainer after this step, 20 minutes of exposure to 10 mg/mL collagenase D plus 10 mg/mL accutase (Gibco) was performed and followed by a 10-minute digestion with 0.05% trypsin/EDTA (Wisent) if necessary. At each stage, dissociated cells were collected by passage through a 40 μm cell strainer. Hepatocytes were excluded from non-parenchymal cell (NPC) preparations by repeated low-speed centrifugations at 50*g* for 5 minutes. NPCs were centrifuged at 350*g* for 10 minutes and the pellet resuspended in ammonium chloride solution to lyse red blood cells. NPCs were finally resuspended in Leibovitz/5% FBS medium, counted, and used either for FACS analysis or for colony-forming assay as described in sections below.

### Flow cytometry analysis

Four million cells were used for flow cytometry preparation and gated based on CD45^–^, CD11b^–^, CD31^–^, MIC1-1C3^+^, CD133^+^, CD26^–^ cell surface markers. Cells were stained with a primary antibody cocktail (biotin-conjugated CD45.2, CD11b, CD31; on ice, 30 minutes) and subsequently washed and incubated on ice for 30 minutes in staining buffer containing streptavidin-conjugated secondary antibody and MIC1-1C3, CD133, and CD26 (Supplemental Table 2). Cells were subsequently resuspended in DAPI-containing buffer. Flow cytometry was performed on a BD Fortessa Analyzer and results analyzed with FlowJo software (Tree Star Inc.). The desired population was obtained through the previously described gating strategy (44).

### Colony-forming assay

Culture dishes (6 cm) were precoated with 2 mL of rat tail collagen I (1 mg/mL 0.1% glacial acetic acid, diluted 1:10 in DMEM), rinsed with PBS before addition of 1.2 × 10^5^ irradiated NIH 3T3 fibroblast feeder cells in 10% FBS DMEM, and allowed to adhere for 24 hours. 1 × 10^5^ NPCs were added (10% FBS, ITS 1×) in DMEM/F12 supplemented with 100 nM dexamethasone, 10 mM nicotinamide, 2.5 mM HEPES, 20 ng/mL human HGF (Life Technologies), 20 ng/mL human EGF (Stem Cell Technologies), and 10 μM ROCK inhibitor (Millipore, Y-27632). Cells were cultured for 12 days at 5% CO_2_ without medium change. For colony characterization, plates were fixed with acetone/methanol (1:1) and stained with Wright-Giemsa stain (pH 6.8). Colonies, defined as organized clusters of at least 10 cells, were scored. Plates used for passaging were trypsinized, and 1,000 cells were plated on a fresh collagen I–coated, 3T3-plated 6-cm dish.

### Immunohistochemistry and immunofluorescence

#### Tissue.

Livers from adults, neonates, and embryos were harvested, fixed overnight at 4°C in 4% PFA in PBS, and embedded in paraffin. H&E and Masson’s trichrome staining was performed by the pathology core at the Toronto Centre for Phenogenomics. Sections (5 μm) of liver tissue were deparaffinized in xylene, rehydrated in a descending concentration of ethanol, treated in Borg Decloaker antigen retrieval solution (pH 9, 5 minutes at 125°C, 10 seconds at 90°C) using a Decloaking chamber (Biocare Medical), and stained using an HRP-AEC kit according to the manufacturer’s instructions (R&D Systems). Sections used for immunofluorescence were treated with antigen retrieval solution (30 minutes at 121°C, 10 seconds at 90°C), permeabilized with 0.2% Triton X-100, and incubated in 1% BSA, 5% normal goat or donkey serum blocking solution for 60 minutes at room temperature before incubation with primary antibodies overnight at 4°C. The slides were then washed in PBS and labeled with fluorophore-conjugated goat or donkey secondary antibodies. Stained sections were mounted using ProLong Gold Antifade Reagent containing DAPI (Life Technologies).

#### Quantifications.

At E16.5, fluorescence intensity quantification of SOX9^+^ periportal nuclei of nascent biliary ducts was performed using ImageJ software (NIH) (mean fluorescence intensity of all SOX9^+^ nuclei per portal vein; ≥9 portal veins per genotype, *n* = 7 embryos).

Adult liver tissue from 8 mice was stained with Masson’s trichrome and scored for portal collagen deposition by digital pathology according to the amount of periportal blue staining (QuPath quantification, https://qupath.github.io), and qualitative inspection of the level of portal triad distortion was assessed by a pathologist. Mice with low levels of collagen and aberration were ranked in the mild group, while others exhibiting strong defects were ranked as severe.

Necrotic tissue post-CCl_4_ was defined by pericentral loss of architecture. These areas were annotated on H&E staining as depicted in Figure 3F and quantified using ImageJ (mean of >20 central veins/necrotic areas per mouse; *n* = 3 mice). Zone 1–2 HNF4α^+^Ki67^+^ proliferating hepatocytes were manually counted using ImageJ software (mean of 3–5 fields of view per mouse; *n* = 3 mice). Pericentral desmin-positive mesenchymal cell migration was assessed by measurement of the fluorescence intensity of desmin fluorescence signal within the necrotic area as shown in Figure 3F (mean fluorescence intensity of ≥7 central veins per mouse; *n* = 3 mice).

#### Colony immunofluorescence staining.

Cells were rinsed at day 18 with 1× TBS before fixation (4% PFA, 10 minutes). The cells were then washed, permeabilized (TBS, 0.025% Triton X-100, 10 minutes), and incubated in blocking buffer (TBS, 10% normal serum) before overnight incubation at 4°C with primary antibody (TBS, 1% BSA). After secondary antibody incubation (TBS, 5% BSA, 2% normal serum, 1 hour at 4°C), a glass coverslip was mounted in the plate using ProLong (DAPI) reagent.

#### Organoid immunofluorescence staining.

Entire Matrigel domes were rinsed with warm PBS and fixed (PBS, 2% PFA, 0.25% glutaraldehyde, 10 minutes at room temperature) before being embedded in Tissue-Tek OCT Compound (Sakura) to preserve organoid morphology. Frozen sections (10 μm) were permeabilized (PBS, 5% serum, 1% BSA, 0.2% Triton X-100, 1 hour at room temperature) before overnight incubation at 4°C with primary antibody (PBS, 5% serum, 1% BSA). After secondary antibody incubation (PBS, 5% serum, 1% BSA, 1 hour at room temperature), a glass coverslip was mounted using ProLong (DAPI) reagent. See Supplemental Table 2 for primary antibody list. *Z*-stack images were obtained using a Zeiss LSM700 confocal microscope and final image composites generated using ImageJ software.

### RNAscope

Detection of *Adam10*, *Adam17*, and *Dlk1* was performed by the STTARR facility at Princess Margaret Cancer Research Tower (Toronto), using the RNAscope Multiplex Fluorescent Reagent Kit v2 (Advanced Cell Diagnostics, catalog 323100) and RNA-Protein Co-Detection Ancillary Kit (catalog 323180), in accordance with the manufacturer’s instructions. Combined RNAscope and immunofluorescence staining sections were processed as for multiplex immunofluorescence staining after the RNAscope protocol using RNAscope Mm-Dlk1 (catalog 405971), RNAscope Mm-Adam10 (catalog 400161), and RNAscope Mm-Adam17 (catalog 479511) probes. Target RNA was detected with TSA Vivid fluorophore 570 (catalog 323272; 1:2,000). Primary antibodies are listed in Supplemental Table 2. *Z*-stack images were obtained using a Zeiss LSM710 confocal microscope and final image composites generated using ImageJ software.

### Quantitative RT-PCR

RNA was extracted from frozen liver tissue or organoid cultures using TRIzol reagent (Invitrogen) and cDNA generated using a first-strand cDNA synthesis protocol (qScript cDNA supermix, Quanta Biosciences). Gene expression was measured using SYBR Green reagent (PerfeCTa SYBR Green Supermix ROX, Quantabio) in a 7900HT Real-Time PCR System (Applied Biosystems). Expression levels were normalized to hypoxanthine-guanine phosphoribosyltransferase (*Hprt*) and the fold change calculated relative to liver samples from WT mice or control conditions using the 2^–ΔΔCt^ method. Primer sequences are provided in Supplemental Table 3 (mouse) and Supplemental Table 4 (human).

### Single-cell RNA sequencing analysis of publicly available liver datasets

Data processing and analysis were performed in the R statistical environment (v4.1.0). Published datasets were imported from the Gene Expression Omnibus database, reformatted if required, and mined (28, 52, 54). The Seurat R package (v4.0.2) was used to normalize, scale, find variable features, and run dimensionality reduction/unsupervised clustering for each dataset. Quality control metrics were implemented based on parameters provided by respective Methods sections. Via the Seurat package, the DimPlot function was used to display uniform manifold approximation and projection (UMAP) of categorical variables (such as cell populations and conditions). Cell population identities were attributed using ScType (https://github.com/IanevskiAleksandr/sc-type) and curated gene lists. VlnPlot was used to depict the expression of genes of interest across different cell types, and FeatureScatter was used to create correlation scatterplots for gene expression (https://github.com/satijalab/seurat). The Nebulosa R package (v1.13.1) was used to determine and visualize gene-weighted density estimations on UMAPs. Scatterplots depicting the proportion of cells expressing genes of interest were generated using the ggplot2 R package (v3.3.5), and cells were colored by identity class. See Supplemental Table 5 for cell population signatures used to generate UMAPs. For the embryonic liver dataset, cell population identities were provided by the authors (27).

### Western blotting

Snap-frozen liver tissue was homogenized with pestle and mortar in RIPA buffer (50 mM Tris-HCl, pH 7.4, 150 mM NaCl, 10 mM EDTA, 1% Triton X-100, 1% sodium deoxycholate, 0.1% SDS) supplemented with EDTA-free protease inhibitor cocktail (Roche), 200 μM Na_3_VO_4_, and 1 mM NaF. The lysates were cleared by centrifugation at 20,817*g* for 20 minutes. Thirty to forty micrograms of protein was loaded on 10%–12% SDS-PAGE, transferred to nitrocellulose membranes, blocked with 5% milk, and incubated with the primary antibodies overnight at 4°C. β-Actin served as a loading control. Solid black lines signify that lanes were run on the same gel but were non-contiguous.

### ELISA

TGF-β1, HGF, and TNF-α ELISA was performed on protein extracted from snap-frozen liver tissue (150 μg, 100 μg, and 200 μg of total protein, respectively) by homogenization in RIPA buffer per the manufacturer’s instructions (R&D Systems, DY1679, DY2207, DY410).

### Statistics

Statistical analysis was carried out using GraphPad Prism 10 software. Data are expressed as mean ± SEM. Comparisons between 2 groups were performed using 2-tailed unpaired Student’s *t* tests or Mann-Whitney test. Two-tailed paired *t* test was used for organoid treatment analysis. Multiple groups were compared by 1-way ANOVA followed by Tukey’s, Bonferroni’s, or Šidák’s multiple-comparison test, or 2-way ANOVA followed by Šidák’s multiple-comparison test, as indicated in each figure legend. *P* values ≤ 0.05 were considered to be statistically significant; *P < 0.05, **P < 0.01, ***P < 0.001 and ****P < 0.0001.

### Study approval

All clinical investigations were conducted according to Declaration of Helsinki principles and in accordance with University Health Network Research Ethics (REB 21-5237). All subjects provided written informed consent prior to participation.

NC3Rs ARRIVE guidelines, Canadian Council for Animal Care guidelines, and protocols approved by the Animal Care Committee of the Princess Margaret Cancer Centre were strictly followed.

### Data availability

All codes used to interrogate and visualize publicly available human and mouse single-cell datasets can be accessed through https://github.com/kazeera/Defamie-et-al-2024. Values for all data points in graphs are reported in the Supporting Data Values file.

## Author contributions

VD designed and performed the majority of the experiments and analyses, and wrote the manuscript. KA performed scRNA-Seq data analysis. S Sarkar and RS participated in organoid culturing and writing of the manuscript. FV performed the digital pathology quantifications. S Saw facilitated flow cytometry analyses. HF maintained the mouse colony. SRN participated in colony formation assays and CCl_4_ injections. PT reviewed and edited the manuscript. OS performed the pathological analysis of mouse hepatic tissues. PDW validated TIMP1 and TIMP3 mouse knockouts. JJK directed the human liver specimen collection. RK directed the study and wrote the manuscript.

## Supplementary Material

Supplemental data

Unedited blot and gel images

Supplemental table 5

Supporting data values

## Version 1 12/19/2024

In-Press Preview

## Version 2 02/03/2025

Electronic publication
