# Supplementary material for: Metalloproteinase inhibitors regulate biliary progenitor cells through sDLK1 in organoid models of liver injury
Source: J Clin Invest. 2024 Dec 19;135(3):e164997. doi: 10.1172/JCI164997 (PMC11785925; doi:10.1172/JCI164997)
Supplement: Supplemental data [file jci-135-164997-s200.pdf]

Defamie et al. Supplemental Figure 1
(accompanies Main Figure 1)

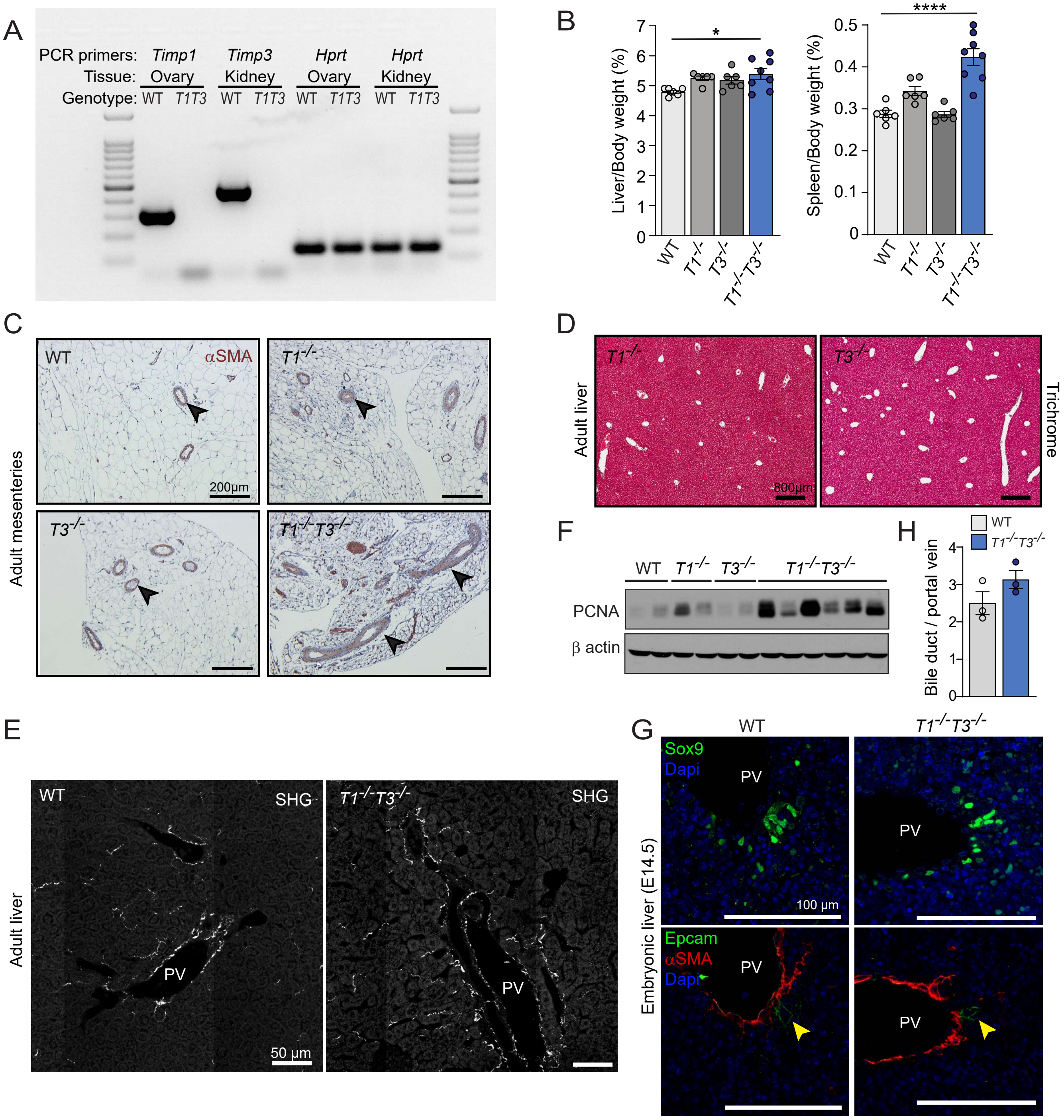

Supplemental Figure 1: Alterations of liver, spleen and mesenteries in *T1−/−T3−/−* mice.
(A) PCR for *Timp1*, *Timp3* or *Hprt* on cDNA from kidney (high in *Timp3*) and ovary (high in *Timp1*) isolated from WT or *Timp1−/−Timp3−/−* (*T1T3*) double knockout mice, absence of a band (PCR product) shows knockout of the gene. **(B)** Liver and spleen weights from 12-week-old mice, n≥6 mice per group. One-way ANOVA with Tukey's multiple comparison test. $^{*}P < 0.05$, $^{****}P < 0.0001$. **(C)** αSMA immunostaining for mesenteric blood vessels (arrowheads) from 12-week-old mice. **(D)** Masson trichrome staining of liver sections for the single knockout *Timp1−/−* (*T1−/−*) and *Timp3−/−* (*T3−/−*). **(E)** SHG images of 12-week-old liver highlighting collagen I fiber (white) in portal vein (PV) vicinity. **(F)** Western blotting of PCNA using liver homogenates from 12-week-old mice, one animal per lane. **(G)** Immunofluorescence showing biliary duct cell commitment in E14.5 embryonic livers as seen by SOX9+ hepatoblasts and Epcam expression (arrowheads) around portal vein. **(H)** Number of bile ducts per portal vein in adult tissue, n=3 livers/genotype (mean of 10 field of view per liver).

Defamie et al. Supplemental Figure 2
(accompanies Main Figure 1)

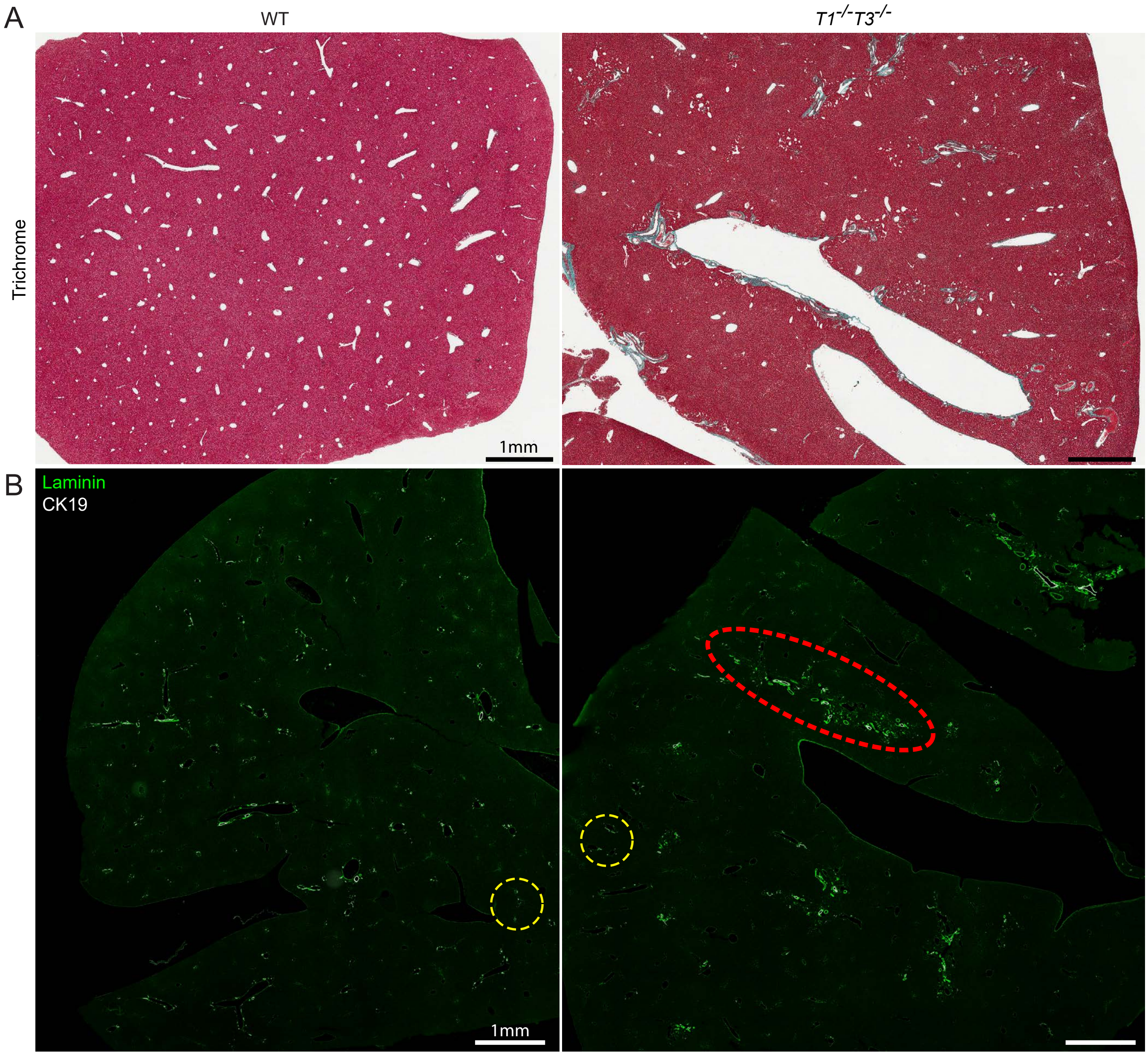

Supplemental Figure 2: Large view of WT and $T1^{-/-}T3^{-/-}$ livers.
(A) Masson trichrome staining and **(B)** immunofluorescence for CK19 and Laminin of hepatic left lobe. Area circled by yellow dashed line depicts normal portal triad while red dashed line highlights structural abnormalities.

Defamie et al. Supplemental Figure 3
(accompanies Main Figures 1 and 3)

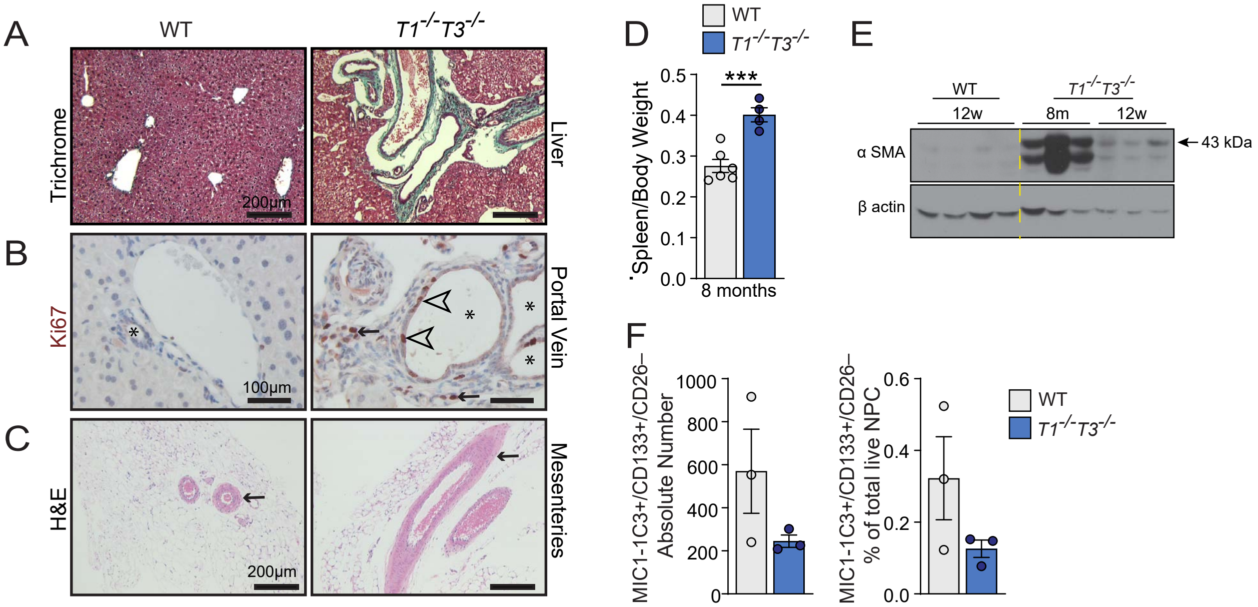

Supplemental Figure 3: Severity of liver defects in aged TIMP deficient mice.
(A) Masson trichrome staining highlights periportal collagen accumulation in blue, severe portal parenchyma and sinusoid dilatation in 8-month-old *T1⁻/⁻T3⁻/⁻* liver. **(B)** Ki67 immunostaining reveals proliferating cholangiocytes (arrowhead) in bile ducts (*) and periportal interstitial cells (black arrow). **(C)** Hematoxylin and Eosin staining for mesenteric tissue, black arrows point to blood vessels. **(D)** Spleen to body weight ratios, n≥4 mice. Tow-tailed Student's *t*-test, ***$P < 0.001$. **(E)** Western blotting of αSMA from liver homogenate, one animal per lane. Yellow dashed line delineates lanes that were run on the same gel but were non-contiguous. **(F)** LPC population in aged *T1⁻/⁻T3⁻/⁻* mice. Flow cytometry analysis of MIC1-1C3+CD133+CD26– LPCs in one-year old WT and *T1⁻/⁻T3⁻/⁻* mice, n=3 per group.

Defamie et al. Supplemental Figure 4
(accompanies Main Figure 1)

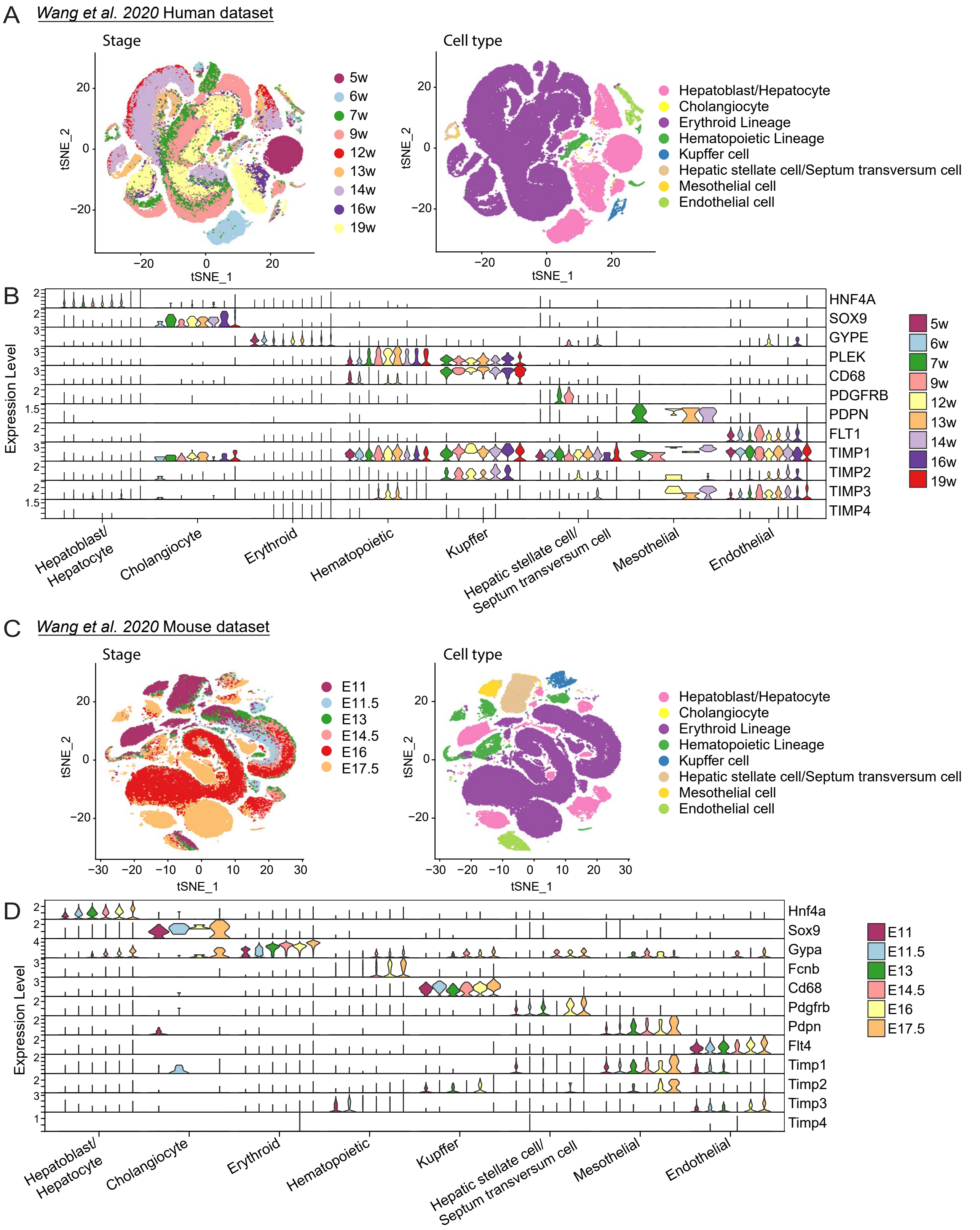

Supplemental Figure 4: Timp expression in human and mouse fetal livers.
ScRNA-seq analysis of fetal human and mouse hepatic cell transcriptomes (>100,000 single cell/species) from Wang et al. 2020 study. **(A)** t-SNE visualization of isolated fetal human hepatic cells showing developmental stages and cell type clusters. **(B)** Violin plot showing the expression of Timps and marker genes for each cell population. **(C)** t-SNE visualization of isolated fetal mouse hepatic cells showing developmental stages and cell type clusters. **(D)** Violin plot showing the expression of Timps and marker genes for each cell population.

Defamie et al. Supplemental Figure 5
(accompanies Main Figure 2)

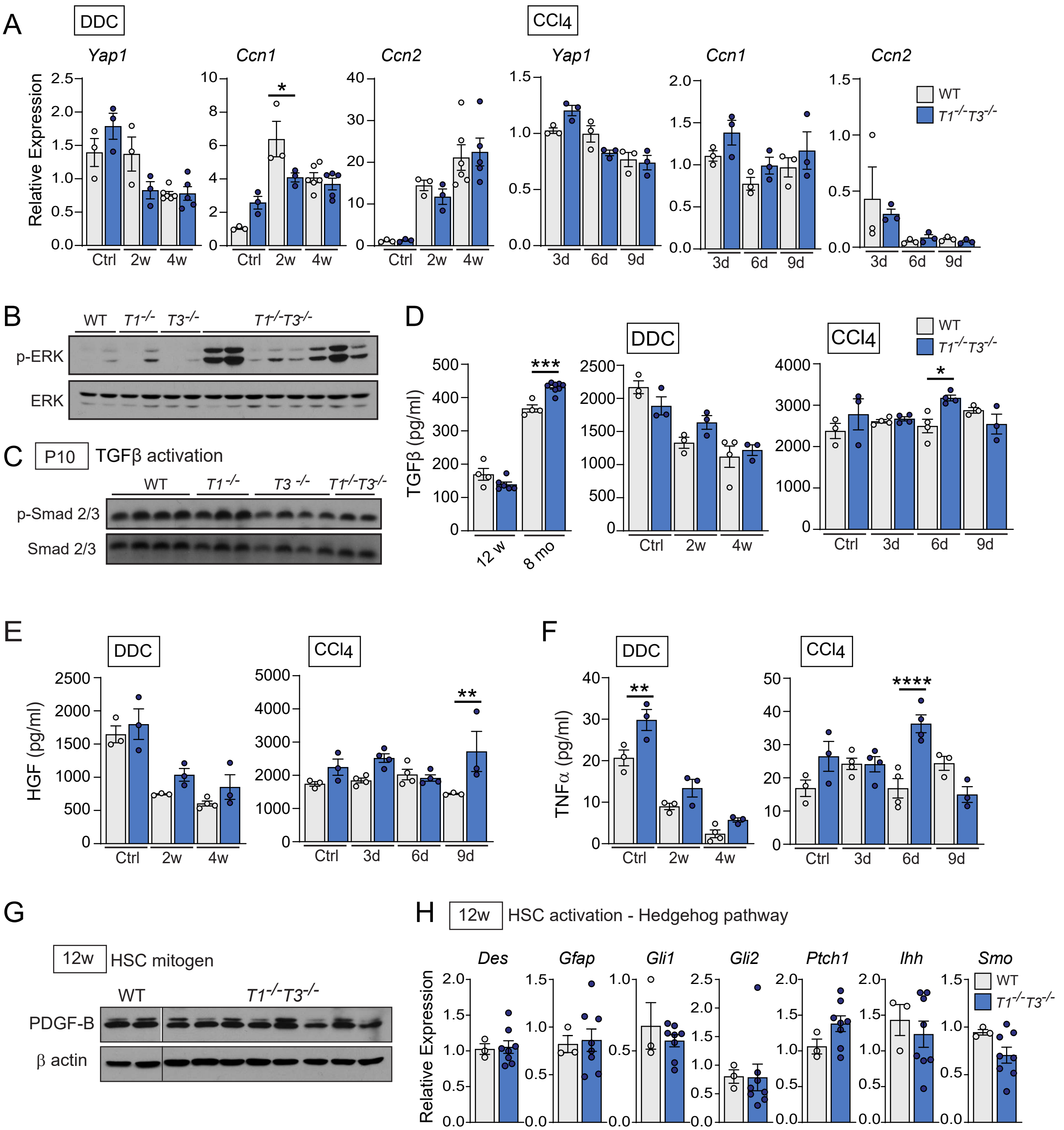

Supplemental Figure 5: Explored molecular pathways *in T1$^{-/-}$T3$^{-/-}$* livers.
(A) Gene expression of *Yap1* and target genes in untreated adult liver and after DDC or CCl_4 treatement. One-way ANOVA with Šídák's multiple comparisons test, n≥3 mice per timepoint. **P* < 0.05. **(B)** Western blotting for phosphorylated ERK and total ERK in adult liver. **(C)** Western blotting for SMAD phosphorylation in postnatal liver (P10). **(D)** ELISA for activated TGFβ in adult, ageing and treated livers. **(E)** ELISA for HGF and **(F)** TNFα in adult hepatic tissue of untreated mice and following DDC or CCl_4 treatements. One-way ANOVA with Šídák's multiple comparisons test, n≥3 mice per timepoint. **P* < 0.05, ***P* < 0.01, ****P* < 0.001, *****P* < 0.0001. **(G)** Western blotting for the hepatic stellate cell mitogen PDGF-B in normal adult liver homogenate. Black line delineates lanes that were run on the same gel but were non-contiguous. **(H)** Relative gene expression for markers of quiescent hepatic stellate cells (*Des*, *Gfap*) and Hedgehog pathway (*Gli1*, *Gli2*, *Ptch1*, *Ihh* and *Smo*) in adult livers, WT n=3; *T1$^{-/-}$T3$^{-/-}$* n=8. For western blots each lane represents one animal.

Defamie et al. Supplemental Figure 6
(accompanies Main Figure 3)

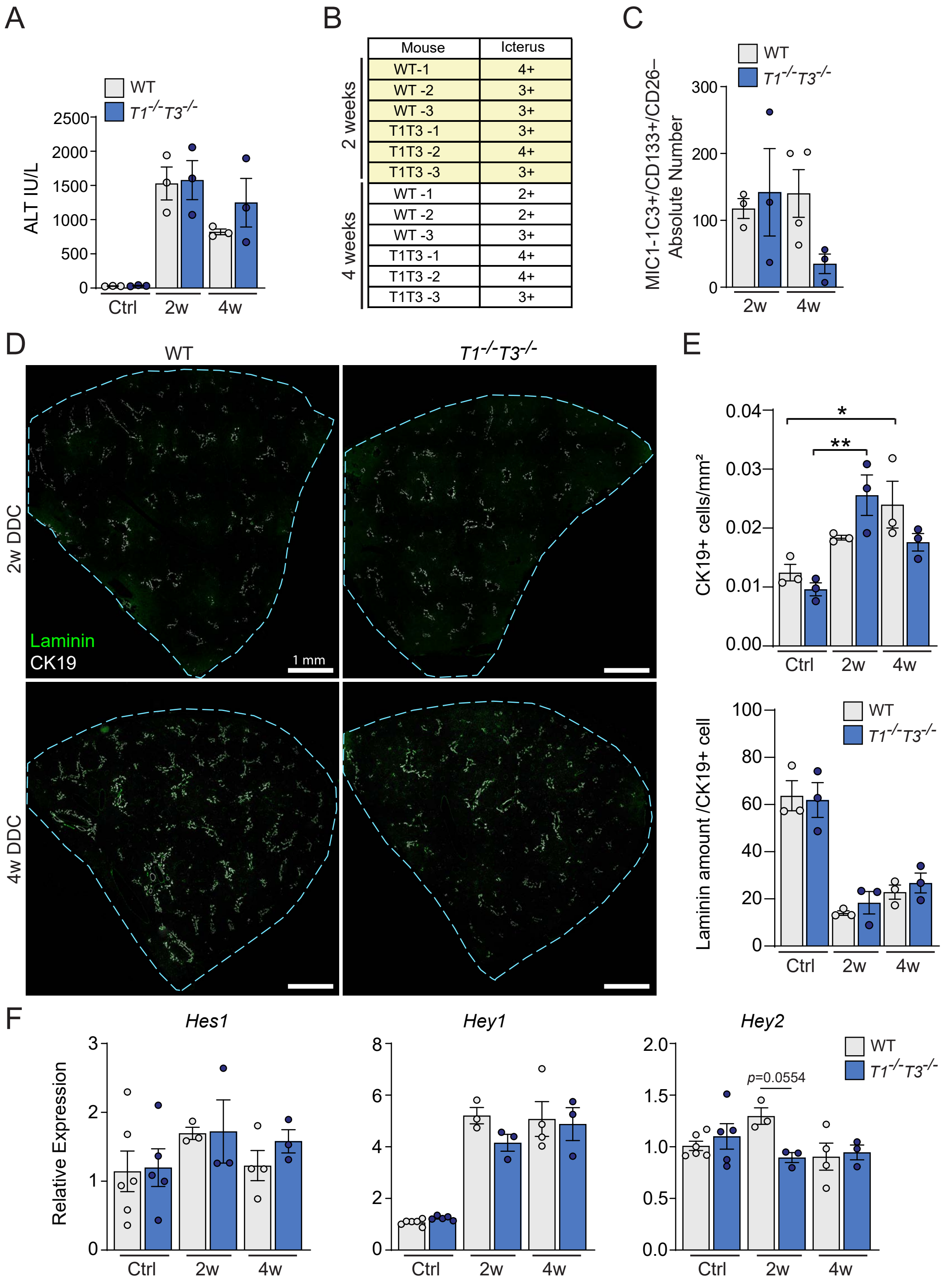

Supplemental Figure 6: Liver response to DDC-induced chronic injury.
Liver damage measure as **(A)** level of serum alanine transaminases (ALT) and **(B)** icterus index, n=3 mice. **(C)** Absolute number of MIC1-1C3+CD133+CD26– cell population, n=3 livers/group. **(D)** Immunofluorescence for laminin and CK19. Dashed blue line delineates the periphery of the left lobe section. **(E)** Number of CK19+ cells per mm² of hepatic tissue and amount of laminin per CK19+ cell in untreated control liver (Ctrl) and after DDC-treatment (mean of 10 ROI/liver, n=3 liver/group). **(F)** RT-PCR for Notch target genes in liver tissue following DDC damage, n≥ 3. One-way ANOVA with Šídák's multiple comparisons test, **P* < 0.05 and ***P* < 0.01.

Defamie et al. Supplemental Figure 7
(accompanies Main Figure 3)

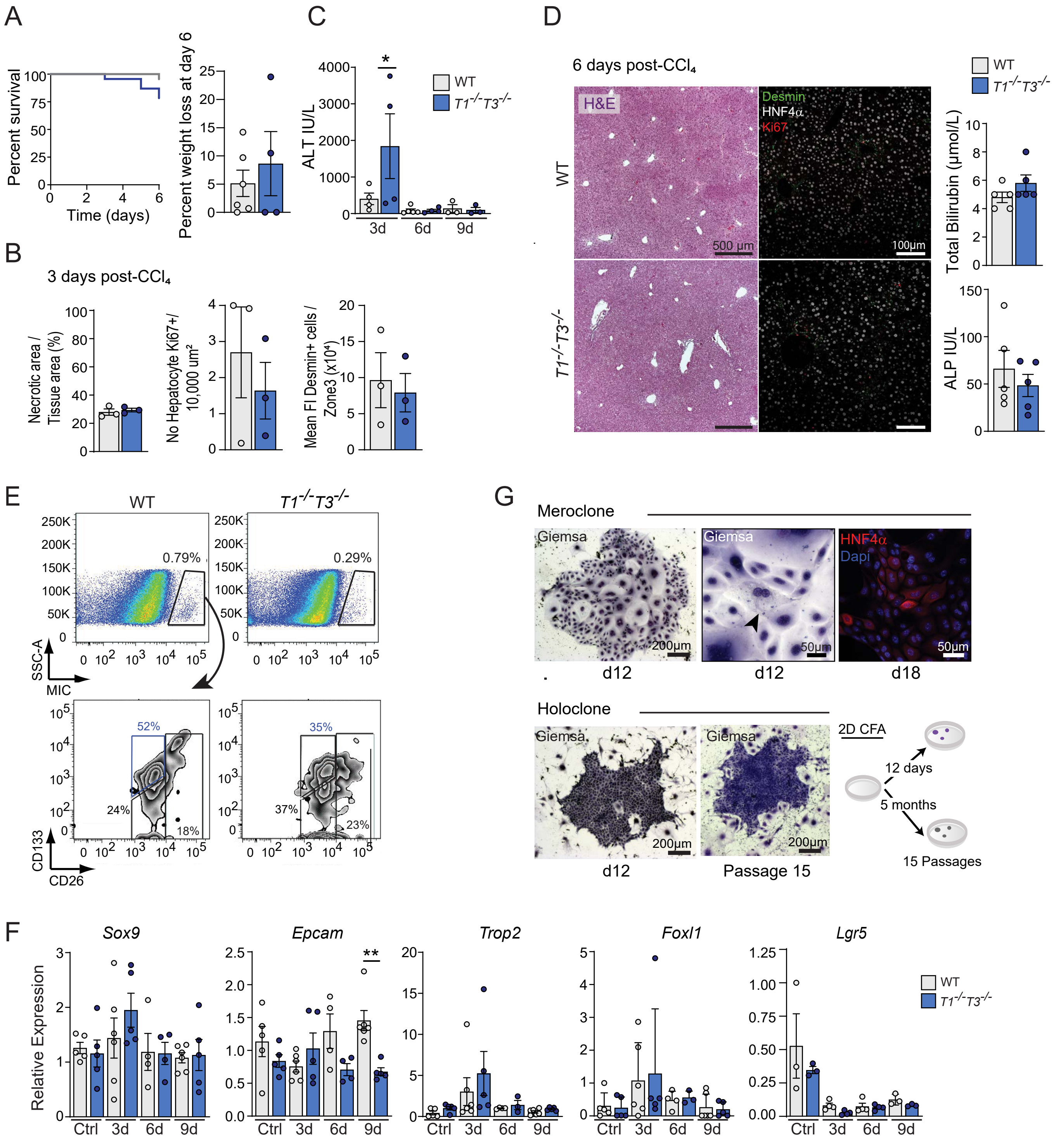

Supplemental Figure 7: Liver response to CCl_4-induced acute injury.
(A) Mouse survival curve (WT n=22; $T1^{-/-}T3^{-/-}$ n=23 animals) and weight loss at day 6 post-CCl_4 (WT n=6; $T1^{-/-}T3^{-/-}$ n=4 mice). **(B)** Measurement of liver damage 3 days post-CCl_4 injection. Quantification of pericentral necrotic area, proliferative zone 1/2 hepatocytes and pericentral mesenchymal cell migration (zone 3 desmin+ FI, fluorescence intensity), n=3 livers/group. **(C)** Level of serum alanine transaminases (ALT) following CCl_4 administration. One-way ANOVA with Šídák's multiple comparisons test, n≥3 mice per timepoint. **P* < 0.05. **(D)** Liver tissue at 6 days post-CCl_4, representative image of hematoxylin and eosin staining (H&E) and immunofluorescence staining for desmin (mesenchymal cells), HNF4α (hepatocytes) and Ki67 (proliferative cells). Level of total bilirubin and alkaline phosphatases (ALP), n=5 mice per genotype. **(E)** Representative flow cytometry profiles of MIC1-1C3+CD133+CD26– LPCs post-CCl_4 insult. **(F)** Gene expression of biliary cell and hepatic progenitor markers from liver tissue extracted 3, 6, and 9 days after CCl_4 administration, n≥3 livers. One-way ANOVA with Šidák's multiple comparison test. ***P* < 0.01. **(G)** Representative brightfield images of meroclone (hepatic epithelial cells; HNF4α immunofluorescence) and holoclone colonies. Representative image of colony type obtained after 15 subsequent passages of primary CFA plate.

Defamie et al. Supplemental Figure 8
(accompanies Main Figure 4)

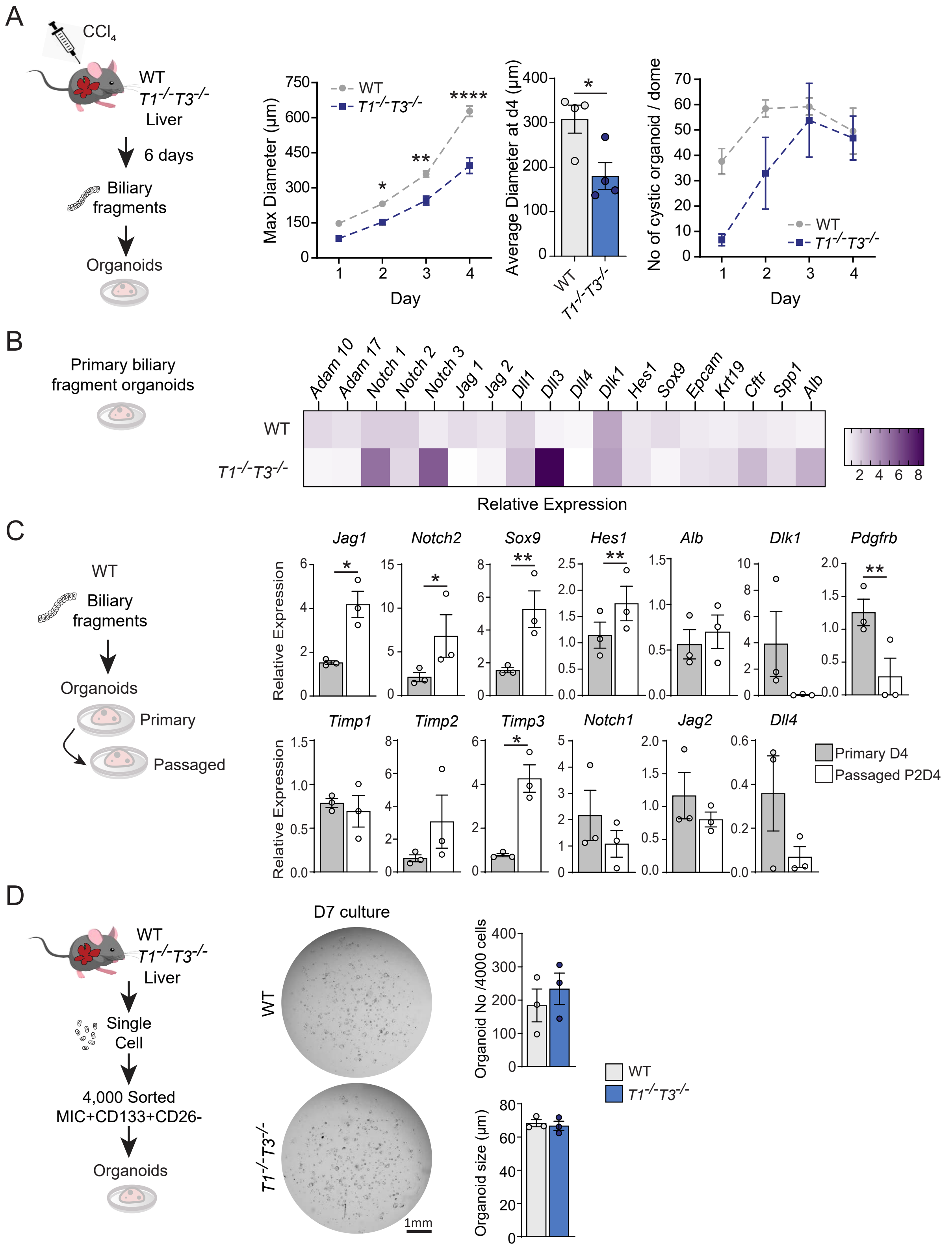

Supplemental Figure 8: Microenvironment dictates LPC fate.
(A) Schematic of organoids derived from CCl_4-treated livers. Maximum diameter and number of organoids per dome during the first 4 days of culture, n=4 livers. Two-way ANOVA with Šidák's multiple comparison test. Average organoid diameter at day 4 culture, two-tailed Student's *t* test. **(B)** Heat map of gene expression in WT and *T1*$^{-/-}$*T3*$^{-/-}$ organoids at day 4 culture, n=4 livers. **(C)** Schematic of WT biliary fragment-derived primary liver organoid and their subsequent passages. Gene expression of Notch pathway components and TIMPs in primary organoids and after 2 passages (n=3), ratio paired *t* test. **(D)** Schematic of the procedure used to derive organoids from 4,000 flow sorted MIC1-1C3+CD133+CD26– biliary cells, n=3 mice per group. Representative Z-stack of brightfield images of an entire dome after seven days in culture. Quantification of the organoid number and mean size. *P < 0.05, **P < 0.01, ****P < 0.0001.

Defamie et al. Supplementary Tables

Supplemental Table 1: Primer sequences used for PCR.

The genomic primers are to genotype mice. The cDNA primers are to demonstrate expression levels.

Genes	Direction	Primers
Timp1 genomic	WT:<br>Common:<br>Neo:	5’-CTCGGACCTGGTCATAAGGGCTAAATTCATGG-3’<br>5’-ACTCTTCACTGCGGTTCTGGGAC-3’<br>5’-CCAAATTAAGGGCCAGCTCATTCCTCCCA-3’
Timp3 genomic	WT:<br>Common:<br>Neo:	5’-AGTTGCAGAAGGCATCCTGGGGATGGCT-3’<br>5’-CAAGAATCTTCTTCTCCCGCTCTCCGCTT-3’<br>5’-CCAAATTAAGGGCCAGCTCATTCCTCCCA-3’
Timp1 cDNA	Forward:<br>Reverse:	5’-AAATGCCGCAGATATGGCCTA-3’<br>5’-TAGTCCTCAGAGCCCACGAG-3’
Timp3 cDNA	Forward:<br>Reverse:	5’-CTTCTGCAACTCCGACATCGT-3’<br>5’-CCACCCTTCTGCCGGAT-3’
Hprt cDNA	Forward:<br>Reverse:	5’-TTGCTGGTGAAAAGGACCTCT-3’<br>5’-TAATGACACAAACTGATTCAAATCCC-3’

Supplemental Table 2: Antibodies used in the study.

Primary antibodies	Suppliers	Dilutions	Product number
**Flow Cytometry**			
CD11b-biotin	ebioscience	1:2000	13-0112-82, clone M1/70
CD133-PerCP-efluor710	ebioscience	1:200	46-1331-82, clone 13A4
CD26-FITC	BD Biosciences	1:200	559652, clone H194-112
CD31-biotin	ebioscience	1:500	13-0311-82, clone 390
CD45.2-biotin	ebioscience	1:500	13-0454-82, clone 104
MIC-1-1C3-Dylight650	Novus Biological	1:100	NBP1-18961
Streptavidin-efluor450	ebioscience	1:200	48-4317-82
**Immunostaining**			
α-SMA	Sigma	1:5000	A2547
Acetylated tubulin	Sigma	1:250	T-6793
CD34	Abcam	1:250	81289
CK19	Abcam	1:30	ab15463
CK19	DSHB	1:500	Troma III
Desmin	Abcam	1:100	15200-1
Epcam	Abcam	1:200	ab71916
Hnf4α	Santa Cruz	1:100	sc-8987
Hnf4α	Santa Cruz	1:100	sc-6556
ki67 (IHC)	Thermo Scientific	1:200	RM 9106-S0
ki67 (IF)	Biolegend	1:200	652402
Laminin	Abcam	1:100	ab11575
Osteopontin	R&D	1:500	AF808
Sox9	Millipore	1:600	ab5535
Vimentin	Abcam	1:100	ab92547
Donkey anti-Mouse AF488	Jackson ImmunoResearch	1:300	715-545-150
Donkey anti-Rat AF647	Jackson ImmunoResearch	1:300	712-605-153
Donkey anti-Rabbit Cy3	Jackson ImmunoResearch	1:300	711-165-152
Donkey anti-Goat AF488	Jackson ImmunoResearch	1:300	705-545-147
Donkey anti-Goat AF647	Jackson ImmunoResearch	1:300	705-605-003
**Western Blots**			
α-SMA	Sigma	1:1000	A2547
b-actin-HRP	Santa Cruz	1:50,000	sc-47778
cleaved Notch 1	Cell Signaling Technology	1:500	#4147
cleaved Notch 2	Millipore	1:1000	07-1234
DLK1	Abcam	1:1000	ab119930
pERK	Cell Signaling Technology	1:1000	#4376
ERK	Cell Signaling Technology	1:1000	#9102
PDGF-B	Santa Cruz	1:100	sc-7878
PCNA	Novocastra	1:500	NCL-PCNA
SOX9	Abcam	1:1000	ab76997
pSMAD 2/3	Cell Signaling Technology	1:1000	#3101
SMAD 2/3	Cell Signaling Technology	1:1000	#3102
HRP-conjugated anti Rabbit	Cell Signaling Technology	1:10,000	#7074
HRP-conjugated anti Mouse	Cell Signaling Technology	1:10,000	#7076
**Neutralizing antibody**			
DLK1 (organoid)	R&D	2 ng/µl	AF8277

Supplemental Table 3: Mouse primer sequences used for quantitative RT-PCR.

Genes	Direction	Primers
Acta2	Forward:<br>Reverse:	5'-TCCTGACGCTGAAGTATCCGATA-3'<br>5'-GGTGCCAGATCTTTTCCATGTC-3'
Adam10	Forward:<br>Reverse:	5'-GCAACATCTGGGGACAAACT-3'<br>5'-TTGCACTGGTCACTGTAGCC-3'
Adam17	Forward:<br>Reverse:	5'-AGGATGCTTGGGATGTGAAG-3'<br>5'-CTGTTTGCTCTGGGAGAACC-3'
Alb	Forward:<br>Reverse:	5'-GTGCCGTAGCATGCGGGAGG-3'<br>5'-GCGCAGATGACAGGGCGGAA-3'
Ccn1	Forward:<br>Reverse:	5'-AGAGGCTTCCTGTCTTTGGC-3'<br>5'-CCAAGACGTGGTCTGAACGA-3'
Ccn2	Forward:<br>Reverse:	5'-AGTGGAGCGCCTGTTCTAAG-3'<br>5'-GTCTTCACACTGGTGCAGCC-3'
Ccnd1	Forward:<br>Reverse:	5'-GGGTGGGTTGGAAATGAAC-3'<br>5'-TCCTCTCCAAAATGCCAGAG-3'
Cd31	Forward:<br>Reverse:	5'-ACGAGAGCCACAGAGACGGT-3'<br>5'-CATGAACAAGGCAGCGGGGT-3'
Cd68	Forward:<br>Reverse:	5'-AGGCCGTTACTCTCCTGCCA-3'<br>5'-TGGAGGTGGTCCAGGGTGAG-3'
Cftr	Forward:<br>Reverse:	5'-GTCGTCTCGGCATTACAACC-3'<br>5'-CCAGTTGTTTGAGCTGCTGT-3'
Des	Forward:<br>Reverse:	5'-TACACCTGCGAGATTGATGC-3'<br>5'-ACATCCAAGGCCATCTTCAC-3'
Dlk1	Forward:<br>Reverse:	5'-CTTTCCAGAGAACCCAGGTG-3'<br>5'-ACGGGAAATTCTGCGAAATA-3'
Dll1	Forward:<br>Reverse:	5'-AGGTTGCTCTGTGTTCTGCC-3'<br>5'-ATGTTGGTCATCACACCCTG-3'
Dll3	Forward:<br>Reverse:	5'-GTTCCCATCACAAGGTCCAG-3'<br>5'-TCCCTGTCTCCACCAGTAGC-3'
Dll4	Forward:<br>Reverse:	5'-GCAATGAATGTATCCCCCAC-3'<br>5'-ATTCTTGCACGGAGAGTGGT-3'
Epcam	Forward:<br>Reverse:	5'-AGGAAGTACACTGGCATTCACC-3'<br>5'-CCGCGGCTCAGAGAGACT-3'
Foxl1	Forward:<br>Reverse:	5'-TGGCTTTGATCTCATTGGATG-3'<br>5'-CATATTGAGCATTTCCGGTGA-3'
Gfap	Forward:<br>Reverse:	5'-AGAACAACCTGGCTGCGTAT-3'<br>5'-CCAGCGATTCAACCTTTCTC-3'
Gli1	Forward:<br>Reverse:	5'-CCTCCTCCTCTCATTCCACA-3'<br>5'-CTCCCACAACAATTCCTGCT-3'
Gli2	Forward:<br>Reverse:	5'-CCCCATCACCATTCATAAGC-3'<br>5'-CTGCTCCTGTGTCAGTCCAA-3'
Hey1	Forward:<br>Reverse:	5'-ACACTGCAGGAGGGAAAGGTT-3'<br>5'-CAAACTCCGATAGTCCATAGCCA-3'
Hey2	Forward:<br>Reverse:	5'-AAGCGCCCTTGTGAGGAAAC-3'<br>5'-GGTAGTTGTCGGTGAATTGGAC-3'
Hnf1b	Forward:<br>Reverse:	5'-CCCAGCAATCTCAGAACCTC-3'<br>5'-AGGCTGCTAGCCACACTGTT-3'

Supplemental Table 3 (continued).

Genes	Direction	Primers
Hprt	Forward:<br>Reverse:	5'-GAGTCCTGTTGATGTTGCCA-3'<br>5'-GCAAATCAAAAGTCTGGGGA-3'
Ihh	Forward:<br>Reverse:	5'-TGACAGAGATGGCCAGTGAG-3'<br>5'-CAATCCCGACATCATCTTCA-3'
Jag1	Forward:<br>Reverse:	5'-CCCACGTGTTCCACAAACATC-3'<br>5'-CCATGGGAACAGTTATTTGGAGA-3'
Jag2	Forward:<br>Reverse:	5'-TCCTCCTGCTGCTTTGTGATC-3'<br>5'-TCAGGCAGGTCCCTTGCA-3'
Krt19	Forward:<br>Reverse:	5'-GTCCTACAGATTGACAATGC-3'<br>5'-CACGCTCTGGATCTGTGACA-3'
Lgr5	Forward:<br>Reverse:	5'-CCTTGGGAATGTGTGTCAAA-3'<br>5'-CAGCGTCTTCACCTCCTACC-3'
Mki67	Forward:<br>Reverse:	5'-AATCCAACTCAAGTAAACGGGG-3'<br>5'-TTGGCTTGCTTCCATCCTCA-3'
Notch1	Forward:<br>Reverse:	5'-GAATGGAGGTAGGTGCGAAG-3'<br>5'-CTGAGGCAAGGATTGGAGTC-3'
Notch2	Forward:<br>Reverse:	5'-GACGTGCTGGACGTGAATGT-3'<br>5'-CAGGTCTGAGCTGCCTCCTC-3'
Notch3	Forward:<br>Reverse:	5'-GAATCTGGAAGACACCCTGG-3'<br>5'-AAGCGTCTCCTGGATGCTG-3'
Pdgfrb	Forward:<br>Reverse:	5'-TGGTATCACTCCTGGAAGCC-3'<br>5'-CCAGCCCTTCTACTGCTGTC-3'
Prom1	Forward:<br>Reverse:	5'-AGGGCAATCTCCTTGGAATC-3'<br>5'-TGGCCCTCTCTACAAAATGG-3'
Ptch1	Forward:<br>Reverse:	5'-ATGCTCCTTTCCTCCTGAAACC-3'<br>5'-TGAACTGGGCAGCTATGAAGTC-3'
Smo	Forward:<br>Reverse:	5'-GTCATTCTCACACTTGGGCA-3'<br>5'-GCAAGCTCGTGCTCTGGT-3'
Sox9	Forward:<br>Reverse:	5'-CTCCTCCACGAAGGGTCTCT-3'<br>5'-AGGAAGCTGGCAGACCAGTA-3'
Spp1	Forward:<br>Reverse:	5'-CACTCCAATCGTCCCTAC-3'<br>5'-AGACTCACCGCTCTTCAT-3'
Timp1	Forward:<br>Reverse:	5'-CATGGAAAGCCTCTGTGGAT-3'<br>5'-CTCAGAGTACGCCAGGGAAC-3'
Timp2	Forward:<br>Reverse:	5'-GTCATTGCTGCCTTCCTCTC-3'<br>5'-AAAGGGGTGAAGAATGGCTT-3'
Timp3	Forward:<br>Reverse:	5'-AAACATCTGCCTGGGTTGAG-3'<br>5'-CAAGCTTCCAGCCAAACTTC-3'
Timp4	Forward:<br>Reverse:	5'-ACCTCCGGAAGGAGTACGTT-3'<br>5'-TTATCTGGCAGCAACACAGC-3'
Trop2	Forward:<br>Reverse:	5'-AATACCTGTGAGCCCATTGC-3'<br>5'-AGAGCAACTGTACATGCCCC-3'
Yap1	Forward:<br>Reverse:	5'-CCCTCGTTTTGCCATGAACC-3'<br>5'-TCCGTATTGCCTGCCGAAAT-3'

Supplemental Table 4: Human primer sequences used for quantitative RT-PCR.

**Genes**	**Direction**	**Primers**
HPRT	Forward:<br>Reverse:	5'-AAGAGCTATTGTAATGACCAGT-3'<br>5'-CAAAGTCTGCATTGTTTTGC-3'
SOX9	Forward:<br>Reverse:	5'-GGAAGTCGGTGAAGAACGGG-3'<br>5'-TGTTGGAGATGACGTCGCTG-3'
