# Supplementary material for: Metalloproteinase inhibitors regulate biliary progenitor cells through sDLK1 in organoid models of liver injury
Source: J Clin Invest. 2024 Dec 19;135(3):e164997. doi: 10.1172/JCI164997 (PMC11785925; doi:10.1172/JCI164997)

Full unedited gel for Figure 2D P10 -NICD1

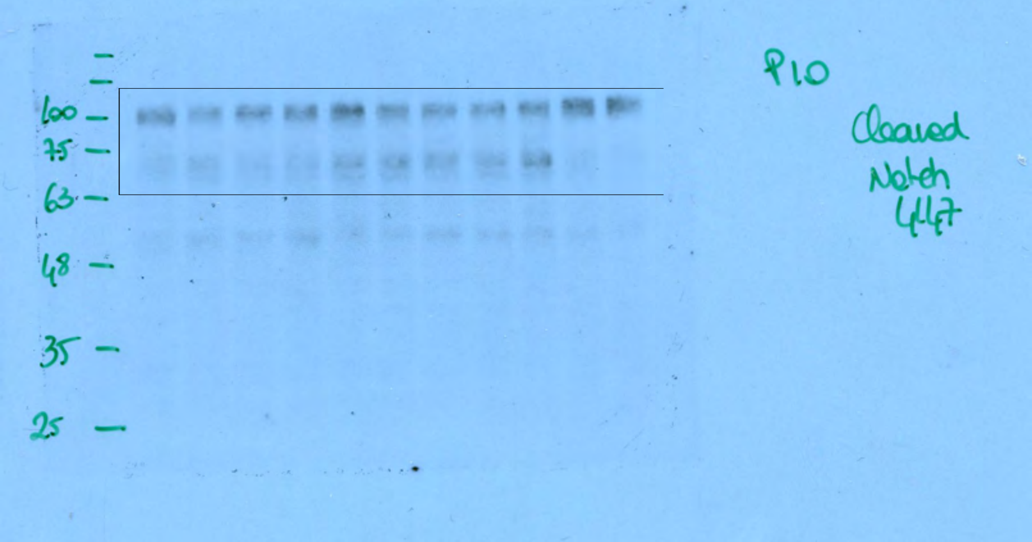

Full unedited gel for Figure 2D P10 -NICD2

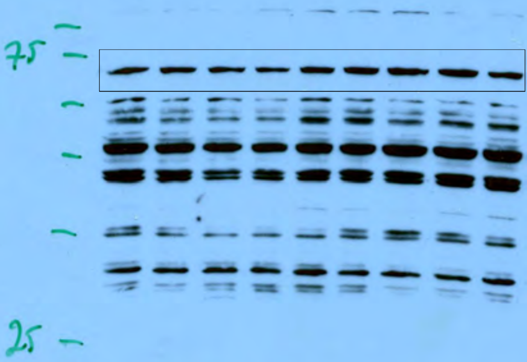

Full unedited gel for Figure 2D - b actin - P10

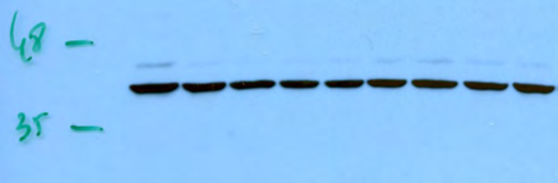

Full unedited gel for Figure 2D - NICD1 - 12w

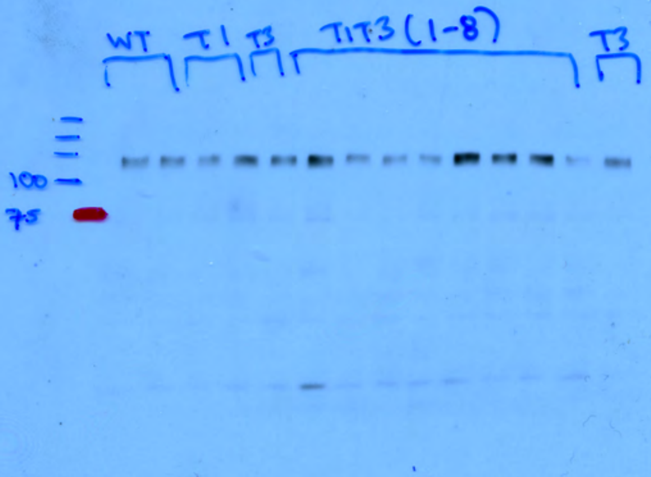

Full unedited gel for Figure 2D - b actin - 12w

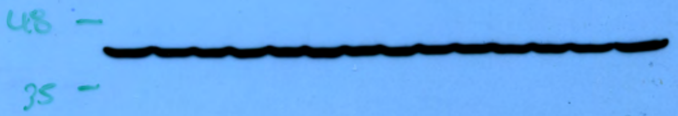

Full unedited gel for Figure 5H- P10- Dlk1

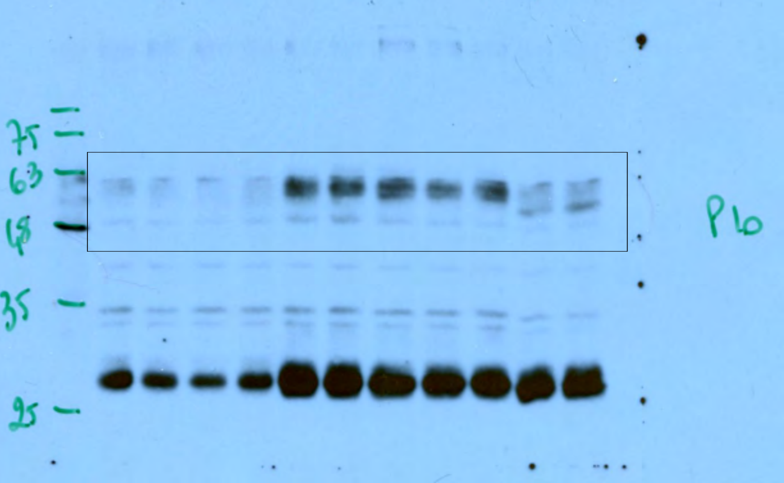

Full unedited gel for Figure 5H-P10-Sox9

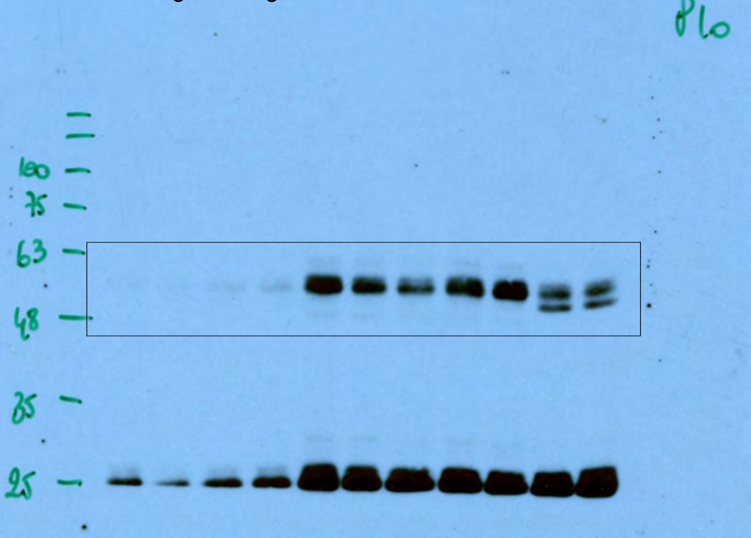

Full unedited gel for Figure 5H-P10-b actin

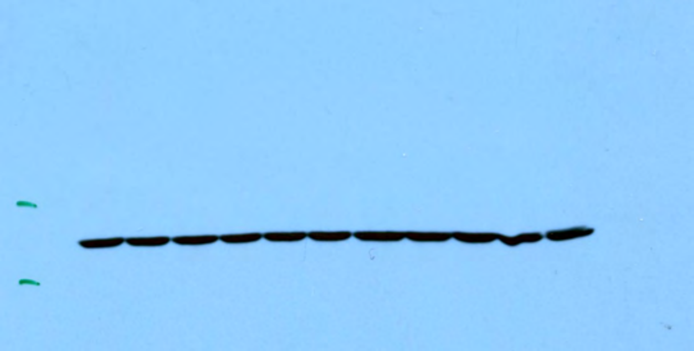

Full unedited gel for Figure 5H-12w-Dlk1

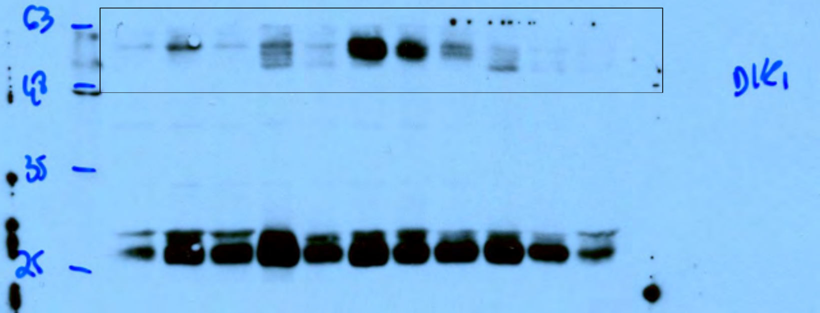

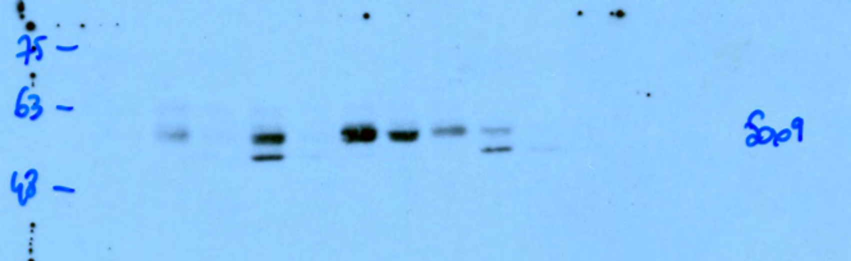

Full unedited gel for Figure 5H-12w-Sox9

Full unedited gel for Figure 5H-12w-b actin

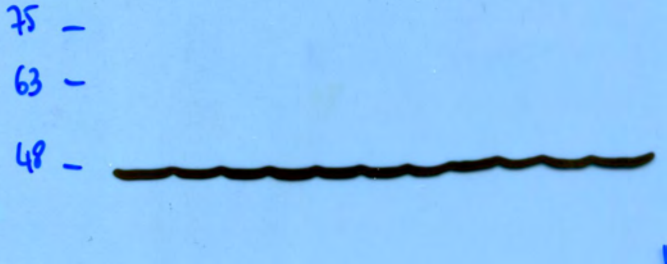

Full unedited gel for Sup Figure 1F - PCNA

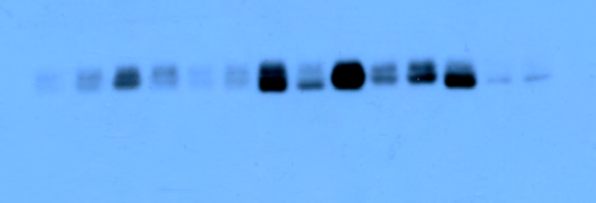

Full unedited gel for Sup Figure 1F - b actin

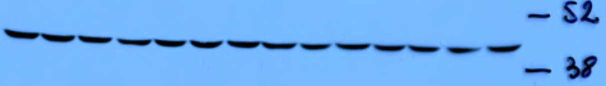

Full unedited gel for Sup Figure 3E-aSMA

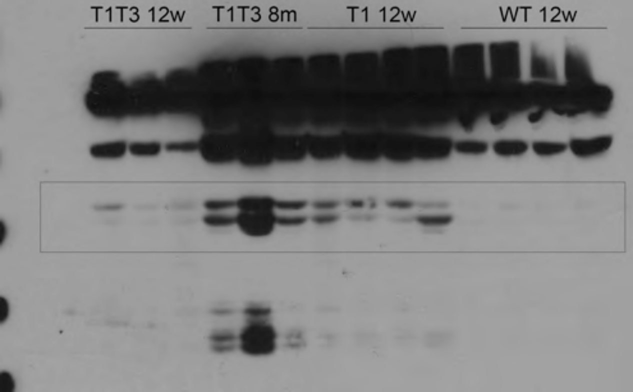

Full unedited gel for Sup Figure 3E-bactin

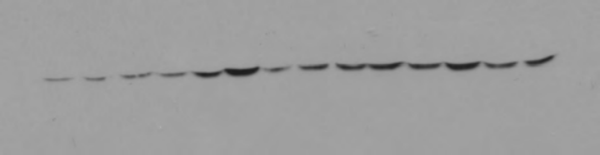

Full unedited gel for Sup Figure 5B - phospho ERK

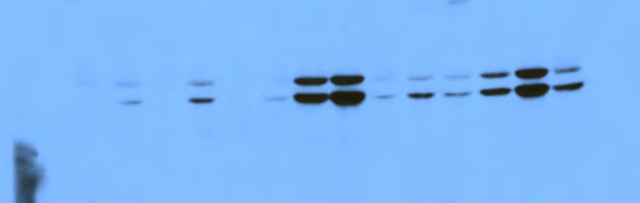

Full unedited gel for Sup Figure 5B - Total ERK

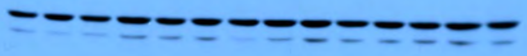

Full unedited gel for Sup Figure 5C - phospho SMAD2/3

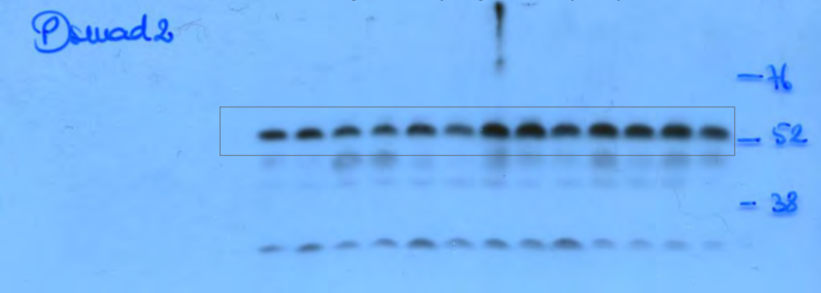

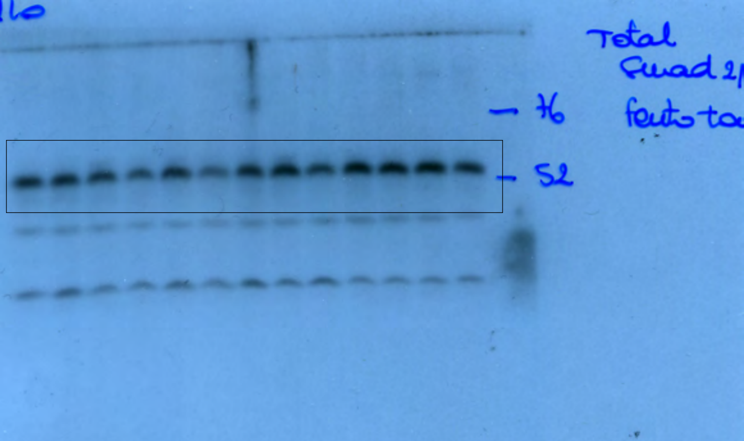

Full unedited gel for Sup Figure 5C - Total SMAD

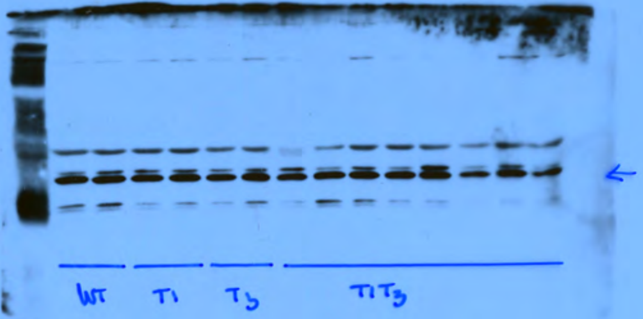

Full unedited gel for Sup Figure 5G - PDGF-B

Full unedited gel for Sup Figure 5G - b actin

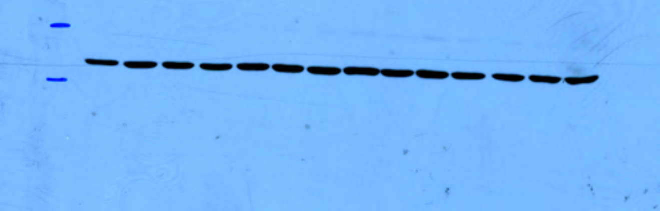

Supplement: Unedited blot and gel images [file jci-135-164997-s201.pdf]
